# The Measurement of Atmospheric Black Carbon: A Review

**DOI:** 10.3390/toxics11120975

**Published:** 2023-12-01

**Authors:** Zhiqing Zhang, Yuan Cheng, Linlin Liang, Jiumeng Liu

**Affiliations:** 1 State Key Laboratory of Urban Water Resource and Environment, School of Environment, Harbin Institute of Technology, Harbin 150090, China; zhangzhiqing950110@163.com (Z.Z.); ycheng@hit.edu.cn (Y.C.); 2 State Key Laboratory of Severe Weather & Key Laboratory for Atmospheric Chemistry, Chinese Academy of Meteorological Sciences, Beijing 100081, China

**Keywords:** black carbon, thermal optical analysis, optical method, laser-induced incandescence method, technical comparison

## Abstract

Black Carbon (BC), the second-largest contributor to global warming, has detrimental effects on human health and the environment. However, the accurate quantification of BC poses a significant challenge, impeding the comprehensive assessment of its impacts. Therefore, this paper aims to critically review three quantitative methods for measuring BC: Thermal Optical Analysis (TOA), the Optical Method, and Laser-Induced Incandescence (LII). The determination principles, available commercial instruments, sources of deviation, and correction approaches associated with these techniques are systematically discussed. By synthesizing and comparing the quantitative results reported in previous studies, this paper aims to elucidate the underlying relationships and fundamental disparities among Elemental Carbon (EC), Equivalent Black Carbon (eBC), and Refractory Black Carbon (rBC). Finally, based on the current advancements in BC quantification, recommendations are proposed to guide future research directions.

## 1. Introduction

**Figure 1:**
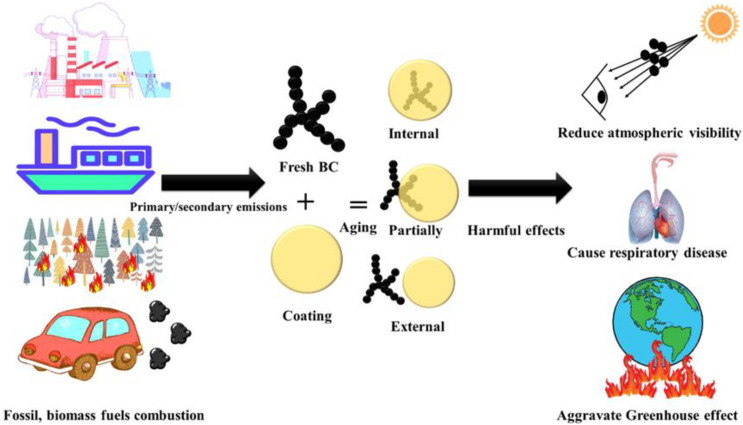
Source, mixed states, and harmful effects of BC aerosol.

Abbreviations and symbols used in this review are summarized in Table 1. Black carbon (BC) is a primary pollutant released from the incomplete combustion of carbon-based fuels and has a relatively short atmospheric lifespan of about a week [1,2,3]. It possesses a microstructure resembling graphite, with the majority of carbon atoms being linked with sp^2^ bonds. This structure exhibits distinct characteristics, including a high sorption capacity for other species (generally > 10 m^2^/g specific surface area), strong light absorption (mass absorption cross-section, MAC > 5 m^2^/g at 550 nm), weak wavelength dependence of light absorption [typical Absorption Ångström Exponent (AAE) of 1–1.5], remarkable thermal stability (volatilization temperature near 4000 K), and insolubility in any solvent [4]. BC originates from various sources such as motor vehicle and ship emissions, open biomass burning (BB), coal combustion, industrial production, and power plants [5,6,7,8,9,10,11,12]. Throughout its atmospheric transport, freshly emitted BC undergoes intricate interactions with other pollutants, resulting in changes to its morphology, size, and composition. This process, known as BC aging, affects its optical and thermal properties, moisture absorption capabilities, and its influence on cloud condensation nuclei and ice nucleation. The duration of this process can span from minutes to days [13,14,15,16,17,18]. Three mixed states of BC exist, namely internal, partial, and external mixed states [19]. The internal mixed state refers to BC being completely enveloped by coating materials, with the polymer center aligning with the coating center, forming a “core–shell” structure. A partially mixed state describes the interface between BC and the coating, while an external mixed state implies that BC and the coating are independent and do not come into contact (Figure 1). BC has adverse health effects, causing respiratory diseases when inhaled and interfering with fetal development when it enters the placenta [20]. Additionally, BC contributes to atmospheric warming by absorbing solar radiation, exacerbating the greenhouse effect globally [21,22]. Melting glaciers in the Himalayas have led to water scarcity affecting 25% of the population within the global catchment area [5]. Therefore, it is imperative for all countries to develop a comprehensive BC emission inventory, implement effective measures to reduce BC emissions, and promptly mitigate the impact of the greenhouse effect [23,24].

In recent years, significant research progress has been made regarding the properties and environmental effects of BC [3,25]. However, the issue of quantifying BC remains a major source of uncertainty. Different detection methods, such as the thermo-optical method and optical method, have been developed based on the distinct physical properties of BC (thermal and optical). These methods often yield substantial differences in results. This uncertainty hampers the comprehensive understanding of BC’s environmental behavior and significantly impedes the comparison of BC observations among researchers [25,26,27,28]. When it comes to unaged BC, the quantitative uncertainties can be largely eliminated. However, when quantifying aging BC, different techniques produce different results. Currently, there are three commonly used quantitative methods for BC: Thermo-Optical Analysis (TOA), the Optical Method, and the Laser-Induced Incandescence (LII) Method. The TOA method operates on the principle of utilizing the divergent thermal and optical properties of organic carbon (OC) and elemental carbon (EC), which evolve sequentially under different temperature and atmospheric conditions. In the Optical Method, the BC mass is determined by measuring the BC light absorption coefficient (b_abs_) and indirectly converting it through the MAC. LII utilizes the refractory properties of BC to rapidly heat it to the gasification temperature (~4000 K), with the mass being determined by the intensity of the incandescent signal [29,30]. The nomenclature for BC varies depending on the quantitative techniques employed. Specifically, BC measured using the TOA is referred to as Elemental carbon (EC), while BC obtained through optical methods is denoted as Equivalent black carbon (eBC). Furthermore, BC measurement conducted via LII is labeled as Refractory black carbon (rBC) [4,28,31].

Measurements of the mass concentration of BC using a single physical property, such as thermal or optical properties, are unreliable. No single technology can comprehensively represent the mass concentration of the BC measurement results. Therefore, it is crucial to carefully evaluate quantitative biases and strive to reduce uncertainty when comparing BC measurements [26,28,32,33]. To achieve this, it is imperative to integrate multiple techniques to assess the consistency and discrepancies in their outcomes, as well as to develop diverse sets of BC data [24,34,35,36]. In recent years, there has been a growing emphasis on BC, encompassing measurement principles [37], sampling techniques [38,39], microstructure [40,41], chemical composition [42], and data quality control [43,44], among others. Nonetheless, there are limited reviews that comprehensively compare quantitative results for BC obtained using different measurement techniques across various atmospheric conditions. Therefore, this paper aims to summarize the principles, applicable contexts, sources of uncertainty, correction approaches, and compare the results of BC measurement technologies. Additionally, it provides suggestions for future research directions regarding BC quantification.

## 2. Elemental Carbon

**Figure 2:**
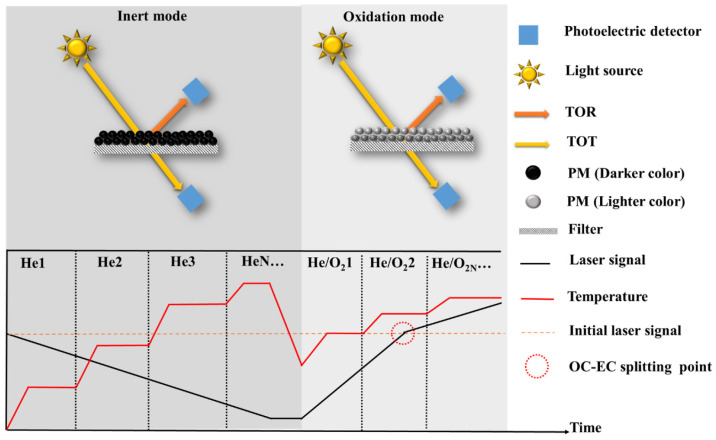
Schematic diagrams and thermogram of TOA.

As the most widely used method for quantifying EC (Figure 2, Table 2), TOA has been extensively employed in long-term atmospheric monitoring networks used by various countries and organizations, including Interagency Monitoring of Protected Visual Environments (IMPROVE), the National Institute for Occupational Safety and Health (NIOSH), the Chemical Speciation Network (CSN), the Speciation Trends Network (STN), the Southeastern Aerosol Research and Characterization network (SEARCH), Aerosol Characterization Experiments (ACE), European Supersites for Atmospheric Aerosol Research (EUSAAR), the Canadian National Air Pollution Surveillance (NAPS), and the California Regional PM_10_/PM_2.5_ Air Quality Study (CRPAQS), among others [45,46,47,48,49,50,51,52,53,54,55]. The principle involves the sequential evolution of OC and EC based on their differing thermal and optical properties under varying temperature and atmospheric conditions. Initially, a PM-containing filter is heated in a furnace, following a predefined thermal–optical protocol. Different protocols consist of specific parameter configurations, with temperature and residence time being the primary variables. The temperature in the inert mode (using He as the carrier gas) is relatively low, facilitating the evolution of OC and the formation of pyrolytic carbon (PC). Subsequently, in the oxidation mode (using He + O_2_ as the carrier gases), higher temperatures are applied to induce the combustion of PC and EC. The temperature program determines the number of heating steps, the temperature at each step, and the corresponding residence time. After the carbonaceous component is gasified, it enters an oxidation furnace together with the carrier gas, where it oxidizes to CO_2_. This resulting CO_2_ is then quantified using Non-dispersive Infrared (NDIR) analysis or further reduced to CH_4_ using a CH_4_ generator, and the generated CH_4_ is quantified using a Flame Ionization Detector (FID) [56]. By analyzing the amount of carbon evolved at different heating stages, the specific content of OC and EC can be calculated [52].

The presence of PC affects the quantitative accuracy of OC and EC by influencing the timing of OC-EC split points. Premature splitting leads to an overestimation of EC and an underestimation of OC, while delayed splitting results in the underestimation of EC and the overestimation of OC [57,58]. Optical correction techniques, such as the thermal optical reflectance method (TOR) [59] for reflected light and the Thermal optical transmittance method (TOT) [60] for transmitted light (T), are employed based on temperature programming to monitor PC formation and evolution, thereby determining the accurate split point between OC and EC. The optical correction principle involves measuring changes in laser signals irradiated on the filter to indicate variations in filter darkness. An increase in darkness causes a decrease in the optical signal, signifying PC formation, while a decrease in darkness corresponds to an increase in the optical signal, indicating PC and/or EC evolution. The moment the optical signal returns to its initial value marks the split point between OC and EC (Figure 2). It is important to note that the accuracy of optical correction relies on two fundamental assumptions: (1) PC evolves before EC, and (2) PC and EC possess identical optical properties. However, in oxidation conditions, PC and EC often evolve simultaneously with distinct optical properties [57,61,62]. Furthermore, during the inert mode, PC and/or EC may prematurely evolve (especially at higher temperatures during the inert phase), while OC may not undergo sufficient evolution and may transition into the oxidation mode (especially with lower temperatures during the inert phase), introducing significant uncertainty in EC quantification [57,58]. These biases are complex and can be attributed to the selection of thermo-optical protocols and chemical components. To address these issues, extensive research has been conducted to investigate the influence mechanisms of thermo-optical protocols and mitigate the interference of non-EC components on EC quantification.

### 2.1. The Effect of Thermo-Optical Protocol on Elemental Carbon Quantification

Currently, the three most commonly utilized thermo-optical protocols are IMPROVE (_A) [52,63], NIOSH [60], and EUSAAR (_2) [46] (Table 3). These protocols exhibit two main differences. Firstly, the inert peak temperature (T_peak_) value of NIOSH is higher compared to that of IMPROVE and EUSAAR. Secondly, NIOSH and EUSAAR specify the duration for each temperature stage, whereas IMPROVE provides general operating principles, such as automatically proceeding to the next step when a distinct carbon peak is formed within the maximum residence time range (Table 3). Other thermo-optical protocols are essentially modified versions derived from the three aforementioned protocols [64]. Due to the numerous thermo-optical protocols available and significant quantitative differences in EC measurements, researchers often face challenges in normalizing results across different protocols. Consequently, many researchers tend to select a single protocol for EC quantification [65]. To address this issue, comparative studies have been conducted in recent years to assess the internal differences of EC among various protocols and investigate the underlying reasons for these discrepancies [53,66,67,68,69].

In general, the Total Carbon (TC) measurements obtained using different temperature programs demonstrate a higher level of consistency compared to EC, with typical deviation ranging from 5–15%. However, it is important to note that the quantitative difference found for EC can reach up to sevenfold, indicating a significant and noteworthy deviation [70]. The influence of the temperature program on EC quantification primarily lies in the selection of the T_peak_. It should be acknowledged, however, that this does not imply that the temperature setting in the oxidation stage does not affect the OC-EC segmentation effect, although its impact is less pronounced than that of the T_peak_. Modifying the T_peak_ affects the relative rate at which various carbon types evolve during TOA, leading to changes in the optically defined OC/EC split and the measured EC. Specifically, an excessively high T_peak_ (e.g., NIOSH) causes the splitting point to appear delayed compared to the actual split, resulting in an underestimation of EC. Conversely, an excessively low T_peak_ (e.g., IMPROVE) may cause OC with high heat resistance to evolve after the split, leading to an overestimation of EC [61,62,71]. For instance, Chow et al. [71] discovered that a higher T_peak_ in the inert mode of NIOSH result in the liberation of O_2_ from mineral oxides, consequently causing early EC evolution. Unfortunately, this particular pre-evolved EC is incorrectly labeled as OC. When this misclassified portion of OC is combined with EC_NIOSH_, a remarkable agreement is achieved with EC_IMPROVE_. Subramanian et al. [62] discovered that, with a T_peak_ of 870 °C, part of the EC evolved earlier during He_4_ (i.e., the fourth temperature gradient of the inert phase), resulting in an underestimation of EC. The amount of EC measured at a maximum temperature of 870 °C during the inert phase (EC_870_) was found to be 20–30% lower than that of EC_700_. Under the T_peak_-550 °C program, some OC evolved after the splitting point, leading to an overestimation of EC (EC_550_ was approximately 50% higher than EC_700_) [62]. Apart from temperature, the optical correction method is another significant factor influencing EC quantification. Cheng et al. [72] confirmed that EC decreases with an increase in T_peak_, and the degree of underestimation also depends on the optical correction method. When using thermal optical transmittance (TOT), EC_580_ was 2.85 ± 1.31 times higher than EC_850_, whereas this difference increased to 3.83 ± 2.58 times when using thermal optical reflectance (TOR). The aforementioned studies highlight that the value of T_peak_ affects EC quantification, and when the same T_peak_ is employed, different optical correction methods can introduce variations in EC quantification. Subsequently, we will delve into the influence of optical correction on EC quantification.

Different types of optical signals exhibit varying sensitivity to the darkness of the filter. In general, reflected light (R) displays a greater sensitivity compared to T. The R is only affected by the PC on the surface of the filter, while the T is affected by the PC on the whole thickness of the filter. Therefore, in the oxidation stage, the R will rise to the initial value before the T; that is, the segmentation point of the thermal–optical reflection method is earlier than that of the thermal–optical transmission method [61,73], resulting in EC_T_ < EC_R_. The magnitude of this difference is contingent upon the temperature program and other chemical components, particularly OC. For instance, in their analysis of environmental PM_2.5_ samples from Hong Kong, Chow et al. [63] consistently observed that EC_T_ (EC defined by transmitted light signal, μg C/cm^2^) was 10% to 40% lower than EC_R_ (EC defined by reflected light signal, μg C/cm^2^) when utilizing the STN protocol. Similarly, Cheng et al. [72] employed IMPROVE_A to investigate environmental PM_2.5_ samples in Beijing, and discovered an average EC_R_/EC_T_ value of 1.50 ± 0.42. Moreover, they found that the disparity between the two ratios was closely associated with secondary organic aerosol (SOA). When SOA/OA < 30%, EC_T_/EC_R_ ≈ 1.0, whereas when SOA/OA > 30%, EC_R_/EC_T_ linearly increased with the rising SOA/OA ratios. Chiappini et al. [74] utilized the IMPROVE method to compare EC levels in European PM_2.5_ samples from rural and urban environments. Significant regional differences in EC_T_/EC_R_ were observed. The EC_T_ value in rural samples was found to be 50% lower than the EC_R_ value; whereas, in urban samples, the difference was only 20%. This phenomenon is believed to be due to variations in the content of light-absorbing organic material, such as brown carbon or humic-like substances, in aerosols between rural and urban areas. TOT and TOR have their own respective applicability. The change in laser signal for TOT covers the entire filter thickness, resulting in a more accurate determination of the splitting point. However, TOT is prone to being affected by TC overloading. On the other hand, TOR exhibits a better detection consistency with different temperature programs compared to TOT, although its accuracy and the precise location of the splitting point are slightly compromised. For example, Brown et al. [75] favor the use of TOT over TOR when employing temperature programs like EUSAAR_2, IMPROVE_A, and NIOSH_870_. This preference stems from TOR’s inability to detect very low levels of EC and meet the required detection limits, particularly at concentrations at which TOR fails to identify small EC values (especially <1 μg C/cm^2^). Moreover, TOR results in uncertainties exceeding 100% at low concentrations, making it unsuitable for routine measurements. However, no specific temperature program has demonstrated superior repeatability and reproducibility across all site types and concentrations when using TOT. Chow et al. [76] prefer TOR measurements because they offer better consistency in EC results across different temperature programs. Additionally, the transmitted light signal is significantly affected by the TC loading of the sample. When testing heavily loaded (dark) samples, the transmitted light signal becomes excessively weak or may even become undetectable. In such cases, the initial transmitted signal is less than 10 counts, which is below the minimum detectable limit (MDL) of the transmittance detector. Hence, it is essential to quantify the differences between EC_T_ and EC_R_ under various temperature programs to identify a more suitable thermo-optical protocol. Subsequently, a temperature program is selected such that the ratio of EC_R_ to EC_T_ is as close to 1.0 as possible.

Given that EC_NIOSH_ consistently yields lower values than EC_IMPROVE_, Zhi et al. [77] attempted to normalize these differences using a regression equation. The equation is expressed as y = (1 − x)/(1 + 4.86x^2^), where x represents the difference factor between EC/TC_IMPROVE_ and EC/TC_NIOSH_ (relative to EC/TC_IMPROVE_). This regression equation helps to alleviate the disparities between EC_IMPROVE_ and EC_NIOSH_. However, it should be noted that this method’s applicability is limited due to its reliance on specific sample types (coal-burning, source, and urban samples). Wu et al. [53] discovered a significant divergence between EC_IMPROVE_ (TOR) and EC_ACE-ASIA_ (TOT) in ambient samples, with EC_IMPROVE_ being 5.4 times higher. This discrepancy can be attributed to variations in temperature programs and optical corrections, with the latter having a more substantial impact. Additionally, a positive correlation was observed between the PC yield and the disparities in EC results across different protocols, indicating that the chemical composition (mainly OC) could influence the level of variation in EC measurements. Therefore, there is currently no standardized transformation scheme for normalizing EC results between protocols. Cavalli et al. [46] introduced a new thermo-optical protocol called EUSAAR_2, which differs from IMPROVE and NIOSH protocols. EUSAAR_2 minimizes the quantitative deviation of EC in ambient samples for three primary reasons. Firstly, compared to NIOSH, EUSAAR_2 extends the residence time in the inert stage, thereby promoting the maximum evolution of OC and minimizing the production of PC. Secondly, in the inert mode, when T_peak_ = 850 °C, more than 20% of LAC (light-absorbing carbonaceous matter) is evolved, whereas at T_peak_ = 550 °C, only 55% of OC is evolved early. To strike a balance, EUSAAR_2 sets T_peak_ = 650 °C, resulting in the minimum evolution of LAC and the maximum evolution of OC in the inert mode compared to other protocols. Thirdly, EUSAAR_2 increases the number of heating steps in the oxidation mode, enhancing the accuracy of the splitting point on the FID curve. Despite the aforementioned advantages, it cannot be concluded that EUSAAR_2 is the universally optimal thermo-optical protocol for all samples. Its suitability depends on the PC yield and the OC-EC segmentation effect. Giannoni et al. [78] discovered that the levels of EC_EUSAAR_2_ and EC_IMPROVE_ in ambient PM_2.5_ samples were 20–40% higher than of EC_NIOSH-like_, and this disparity was independent of the season or sampling location. Cheng et al. [66] reported the following ratios for ambient samples: EC_IMPROVE-A_/EC_NIOSH_ = 1.36 ± 0.21 and EC_IMPROVE-A_/EC_EUSAAR_ = 0.91 ± 0.10. Additionally, the ratios of OC/EC_NIOSH_ to OC/EC_IMPROVE-A_ were 1.43 ± 0.25, and OC/EC_EUSAAR_ to OC/EC_IMPROVE-A_ were 0.89 ± 0.13. These observations indicate that the compatibility between the IMPROVE and EUSAAR methods is superior to that between IMPROVE and NIOSH. This order of EC determination, with EC_IMPROVE-A_ > EC_EUSAAR_ > EC_NIOSH_, is further supported by Brown et al. [75]. Based on the aforementioned research findings, it becomes apparent that achieving uniformity in EC results across different thermal–optical protocols solely through analytical methods or regression equations is challenging. The primary obstacle stems from the substantial variation in the chemical composition of BC aerosols originating from diverse sources, particularly in terms of OC content. A potential solution to enhance reliability involves effectively eliminating OC prior to quantifying EC through TOA [79].

### 2.2. The Effect of Chemical Composition on Elemental Carbon Quantification

Table 4 provides a summary of the mechanisms through which chemical components impact EC quantification [58,80,81,82,83,84,85,86]. Brown carbon (BrC), a light-absorbing component of OC, exhibits strong light absorption at short wavelengths (UV-near visible) [87,88,89]. Its common sources include BB and fossil fuel combustion. BrC has two primary effects on EC quantification: (1) It causes interference with laser signal changes due to its light absorption properties. (2) Certain highly oxidized organic compounds (humic-like substances, HULIS) within BrC possess significant thermal stability and tend to evolve during oxidation processes. Schauer et al. [51] confirmed the temperature program’s varying sensitivity on different PM types, leading to the segmentation effect of OC-EC. Among the samples, wood smoke samples exhibited the highest sensitivity to temperature program changes in the EC results, followed by ambient and fly ash samples. Black carbon and SOA samples displayed the least sensitivity. Reisinger et al. [90] established a correlation between the proportion of BrC in LAC and the quantitative differences in EC obtained from different thermo-optical protocols. Similarly, Cheng et al. [91] reported an overall 80% lower EC_NIOSH_ concentration in ambient samples compared to EC_IMPROVE_. This disparity exhibits noticeable seasonal and regional patterns. In particular, the difference is more pronounced during spring relative to winter, and coastal and rural areas show larger gaps compared to urban regions. This can be attributed to the substantial release of BrC from biomass burning during spring, as well as the higher abundance of SOA in coastal and rural areas compared to urban regions [91]. To mitigate the interference caused by BrC and improve the accuracy of OC and EC differentiation, optical correction techniques involving the utilization of a He-Ne laser (which is minimally absorbed by BrC at red wavelengths) or multi-wavelength lasers have been proposed [92,93].

The Carbonate carbon (CC) content in ambient PM_2.5_ is typically less than 5%, rendering its effect negligible. However, in areas prone to dust, the CC content in PM10 can reach up to 55% [94]. During the inert high-temperature stage, CC undergoes transformations that can impact the carbon peak signal, with the evolution temperature varying depending on the sample type. For instance, calcium carbonate samples decompose at an inert temperature of 550 °C [71], while natural calcite can be decomposed at an inert temperature of 650 °C [46,80]. To mitigate the influence of CC, one approach is to heat the filter samples in O_2_ at 460 °C for 60 min, thereby eliminating OC and EC. This rapid method allows for the determination of CC in coarse particles (PM_2.5_–PM_10_ μm) and is suitable for monitoring a large number of samples, such as daily samples collected using high and low volume samplers [95]. Alternatively, fumigating the filter with HCl can also be employed to remove CC, achieving a removal efficiency of up to 99% [80].

Metals are often present in aerosols near railway tracks, subways, and mines. Metal oxides can release O_2_ during the inert high-temperature stage (e.g., Fe_2_O_3_ releases O_2_ at 850 °C under inert conditions), promoting the early evolution of EC [83]. Metal salts lower the oxidation temperature of EC in diesel PM and enhance the pyrolysis of OC, resulting in an 80% underestimation of EC (which, in some cases, can be overestimated by 40%, depending on the metal-to-carbon mass ratio). Among these, transition metals (CuCl_2_, FeCl_2_, FeCl_3_, CuCl, ZnCl_2_, MnCl_2_, CuSO_4_, Fe_2_(SO_4_)_3_) exhibit a greater influence compared to alkali metals (NaCl, KCl, Na_2_SO_4_) and alkali earth metals (MgCl_2_, CaCl_2_). Copper and iron chlorides have a more significant impact than sulfates [84]. Inorganic salts can alter the temperature and pyrolysis degree of carbonaceous components. Novakov and Corrigan [85] discovered that the combustion temperatures of EC and OC (relatively nonvolatile and having a similar combustion temperature to EC) in biomass combustion smoke particles primarily depend on the Na and K content. Na and K are believed to catalyze carbon combustion, leading to a reduction in the combustion temperature of the aforementioned carbon components by over 100 °C. Yu et al. [58] observed a significant impact of NH_4_HSO_4_ on the pyrolysis degree of OC. In the presence of NH_4_HSO_4_, the PC generated from starch and cellulose exhibited two to three times higher yields compared to reactions without NH_4_HSO_4_. However, the presence of NH_4_HSO_4_ led to a 15% decrease in levoglucosan-derived PC production. Refractory oxygen-containing surface groups (CO_1_^+^ and CO_2_^+^ fragments) in diesel engine exhaust PM also contribute to the premature evolution of EC under inert conditions [86].

Thus, it becomes apparent that employing a single thermo-optical protocol for detecting various sample types is impractical. To achieve relatively accurate measurements of EC, it is essential to utilize a minimum of two thermo-optical protocols and carefully ensure result consistency.

### 2.3. Solvent Extraction Method

To mitigate the output of PC, researchers have employed various strategies. For instance: (1) Optimizing the thermo-optical protocol: Cavalli et al. [46] modified the NIOSH protocol by adjusting the T_peak_ size, number of heating steps, and residence time to maximize OC evolution in the inert mode and minimize premature LAC evolution. (2) Developing a new thermo-optical protocol: Zhang et al. [96] introduced a four-step TOA protocol that incorporates different temperature gradients before the inert mode and introduces O_2_ to eliminate OC. (3) Employing the drying method: Lappi and Ristimäki [97] utilized CaSO_4_ in room temperature or high-temperature air (180 °C) to reduce water, sulfuric acid, and most volatile organic compounds (VOCs) in the sample. (4) Utilizing solvent extraction: Cui et al. [98] employed either ultra-pure water or organic solvents to eliminate non-BC substances in PM. Ultra-pure water can remove sulfates, nitrates, ammonium salts, and water-soluble organic carbon (WSOC), while organic solvents can extract insoluble organic carbon (ISOC). Among these methods, solvent extraction (Table 5) offers numerous advantages [62,78,97,98,99,100,101,102,103,104,105,106,107,108,109,110].

For instance, the removal effect of OC is noticeable, leading to a significant reduction in the PC yield of the treated samples. This treatment has also resulted in a substantial improvement in the accuracy of EC quantification. Several studies have reported similar findings [62,78,99,100,106,111]. In a recent study by Haller et al. [40], it was observed that the laser signals from water extraction samples (ambient samples) did not show a significant decrease in TOA, but exhibited a substantial decrease when analyzing untreated samples. This indicates that the water extraction process effectively removes WSOC from the samples, thereby reducing the PC yield. Similarly, Subramanian et al. [62] employed a mixture of dichloromethane, acetone, and hexane for extracting ambient PM_2.5_ samples. By comparing the thermal spectra of the samples before and after solvent extraction, the authors noted that although solvent extraction did not completely inhibit the formation of PC, it managed to reduce PC generation by 81%.

Particle loss is inevitable during the solution extraction process. However, analyzing the thermo-optical spectrum of the sample before and after treatment can effectively indicate whether the original sample’s EC content has been overestimated or underestimated. Additionally, treatment can enhance the consistency of EC detection results across thermal–optical protocols. Piazzalunga et al. [100] reported a strong correlation (R^2^ > 0.87) in the EC results obtained from three protocols [inert peak temperature 870 °C (He-870), EUSAAR_2, and inert peak temperature is 580 °C (He-580)] for both untreated and washed samples. After filter washing, the EC disagreement between EUSAAR_2 and He-870 decreased from 1.49 to 1.24 (−17%). Similarly, the disagreement between EUSAAR_2 and He-580 reduced from 1.59 to 1.42 (−11%). Furthermore, the study revealed that EC concentrations were generally higher in washed samples compared to untreated samples, with increases up to 54% (He-870), 24% (EUSAAR_2), and 43% (He-580) when measured against the EC values obtained from untreated filters. This suggests that measurements of untreated filters may result in an underestimation of EC levels.

Giannoni et al. [78] employed the He-870, He-550, and EUSAAR_2 protocols to analyze ambient PM_2.5_ samples both before and after water extraction. In the case of untreated samples, EC_EUSAAR_2_ and EC_He-550_ exhibited a 20–40% higher value compared to EC_He-870_. However, this discrepancy was mitigated in the water extraction samples. Specifically, the difference between EC_EUSAAR_2_ and EC_He-870_ was reduced to less than 10% following the removal of WSOC, which constituted approximately 28–55% of TC, through water extraction. An improvement in measurement consistency among protocols was observed. Through a comparison of EC results from untreated and washed samples, the authors determined that the He-870 protocol was more suitable than the other two thermo-optical protocols, as it provided a better consistency in EC measurements before and after washing (EC_untreated_/EC_washed_ = 0.88–1.01). Cheng et al. [110] reported a decrease of 84% and 88% in PC_IMPROVE_ and PC_NIOSH_, respectively, in ambient PM_2.5_ samples after methanol extraction. Conversely, EC_IMPROVE_ and EC_NIOSH_ increased by 45% and 110%, respectively. Methanol extraction also enhanced the consistency of EC quantification between the two protocols, with EC_IMPROVE_/EC_NIOSH_ changing from 1.71 ± 0.31 in the untreated sample to 1.16 ± 0.10 post-extraction. Liu et al. [86] reached a similar conclusion, observing that, after methanol extraction of ambient PM_2.5_ samples, EC_IMPROVE_ and EC_NIOSH_ increased by 24% and 62%, respectively, resulting in a decrease in the ratio of EC_IMPROVE_ to EC_NIOSH_ from 2.24 ± 0.31 to 1.65 ± 0.14. The transmitted light attenuation [ATN = ln(I/I_0_), where I_0_ represents the incoming light intensity and I is the light intensity after passing through the filter] has a clear linear relationship with EC loading. It is assumed that the increase in ATN is solely due to light absorption by EC which accumulates on the filter, and the EC concentration is calculated based on the rate of change of attenuation. However, when EC exceeds a certain threshold, the change in ATN becomes less noticeable (i.e., ATN saturation), leading to a potential underestimation of EC. To address this issue, solvent extraction methods can effectively reduce such uncertainty [86]. In their study, they employed methanol for extracting heavily polluted ambient samples, which resulted in extracted samples that did not exhibit ATN saturation. There was a significant reduction in deposited OC (85%), thereby greatly reducing the uncertainty of EC quantification caused by PC, improving the linear relationship between ATN and EC loading.

In summary, the solvent extraction method offers several advantages: Firstly, it effectively eliminates interfering substances such as inorganic salts and OC, especially when employing the two-step extraction method. Secondly, it efficiently mitigates the impact of the ATN saturation effect, particularly in heavily contaminated samples. Thirdly, it successfully alleviates the interference of PC in EC quantification. Fourthly, it significantly improves the consistency of EC quantification across different thermo-optical protocols. Lastly, it highlights the advantage of measurement consistency before and after extraction using specific thermo-optical protocols. Therefore, we consider solvent extraction as an indispensable step preceding EC quantification.

## 3. Equivalent Black Carbon

The optical method is an indirect technique used to measure the mass concentration of BC [112]. It involves determining the eBC by measuring the b_abs_ of light-absorbing carbon (LAC), and then applying the conversion factor MAC (Mass Absorption Cross-section, C = b_abs_/MAC). Based on the measurement principle, optical methods can be categorized into in situ [113,114,115,116,117] and filter-based techniques [118,119,120,121,122] (Table 6). In general, when detecting PM with fewer non-BC impurities (e.g., freshly emitted soot particles), a high level of agreement in b_abs_ measurements can be achieved across different optical instruments. However, the consistency of b_abs_ measurements tends to decrease when the PM contains a high content of non-BC impurities (e.g., forest fire smoke particles), as it is influenced by various bias effects. Instrument manufacturers often provide a fixed value for MAC, which introduces the most significant uncertainty in the optical method. The presence of non-BC substances (such as mine dust, BrC, secondary inorganic salts) within aerosols leads to modifications in the MAC of aerosols, resulting in an equivalent amount that deviates from pure BC b_abs_ but approximates it closely. The degree of change depends on BC’s chemical components, mixing state, and morphological characteristics [4]. For instance, internally mixed BC (with BC as the core) exhibits a significant enhancement in light absorption. Freshly emitted BC has a MAC value of 7.5 ± 1.2 m^2^/g at 550 nm, which increases to 15 m^2^/g when it is mixed with other components [13,27]. Therefore, accurate measurement of eBC requires assuming that the aerosol contains only one light-absorbing substance, namely BC, and that non-BC components do not impact the MAC of BC. However, natural aerosols often contain multiple light-absorbing or light-scattering non-BC substances. Consequently, it becomes crucial to precisely quantify the contribution of non-BC components and their mixing states to BC’s optical properties in order to determine the actual MAC of BC [15,16,123]. Moreover, we can also eliminate the interference of volatile non-BC substances by heating the samples [124,125].

### 3.1. Filter-Based Technique

The filter-based technique originates from the discovery made by Rosen et al. [128] that the level of ATN is directly proportional to the concentration of graphite soot particles. Since then, several researchers have further refined and optimized this method, which is now widely employed for measuring aerosol b_abs_ [34]. This technique offers various advantages such as simplicity in operation, cost-effectiveness, insensitivity to gaseous interferences, and suitability for field measurements. However, it is important to note that several biases can potentially result in the overestimation of b_abs_ values.

The measurement principle is to indirectly quantify the b_abs_ of deposited PM using the amount of ATN of the laser through the filter [129]. According to the Beer–Lambert law:

(1) $$I=I_{0}e^{-b_{pf}x}$$

(2) $$b_{pf}=\frac{A}{V}\frac{\ln\left(\frac{I_{0}}{I}\right)}{∆t}$$

where *I*_0_ is the light intensity before transmission, *I* is the light intensity after transmission, *b_pf_* (m^−1^) is the b_abs_ produced by PM and filter, *x* is the thickness of the filter (m), *A* is the collection area of the filter (m^2^), *V* is the velocity of the gas passing through the filter (m^3^/s), and ∆*t* is the sampling time (s). The accuracy of *b_pf_* is based on the fact that the change in laser intensity is only caused by the light absorption effect of the filter and PM. Still, the multiple scattering effects of the filter and the loading effect of PM would cause the measured value of b_abs_ to be greater than the actual value. The filter exhibits the multiple scattering effect [130], in which a light beam passing through the filter scatters in various directions, resulting in a significant decrease in transmitted light intensity. This reduction leads to an overestimation of b_abs_. The multiple scattering effect is influenced by both the filter material and the instrument type used. Conversely, the loading effect of PM [131] refers to the phenomenon where the accumulation of PM on the filter causes particle blockage and reduces ATN. Consequently, this effect leads to an underestimation of b_abs_. The extent of underestimation depends on the level of loading and the optical properties of the deposited PM. Additionally, the PM scattering effect occurs when certain PM components on the filter scatter incident light in all directions, increasing the reflectivity and raising ATN. This results in the overestimation of b_abs_, which is determined by the shape, size, and chemical composition of the PM. However, this bias effect can be disregarded as it is much smaller compared to biases caused by other effects [132].

Currently, researchers have proposed various correction schemes to address the aforementioned bias effects (Table 7) [131,133,134,135,136,137]. However, the multitude of calibration formulas complicates the selection process for operators. Some scholars have even made further advancements and optimizations to existing correction schemes in an attempt to achieve more accurate results. For instance, Kim et al. [130] employed the linear regression line (LRL) method instead of the traditional ratio correction method (i.e., uncorrected data/reference instrument data) to mitigate measurement artifacts. This illustrates the absence of a consensus within the academic community regarding the b_abs_ correction scheme for filter-based technology. Apart from selection of calibration schemes, specific sampling parameters such as humidity, pressure, and temperature can introduce biases into the measurement results. For example, in the Amazon Basin, the presence of liquid organic particles can alter the light scattering effect on the filter surface, thus affecting the sensitivity of Particle Soot Absorption Photometer (PSAP) measurements when the relative humidity (RH) ranges between 20% and 30%, and the temperature is between 24 °C and 26 °C [135].

**Figure 3:**
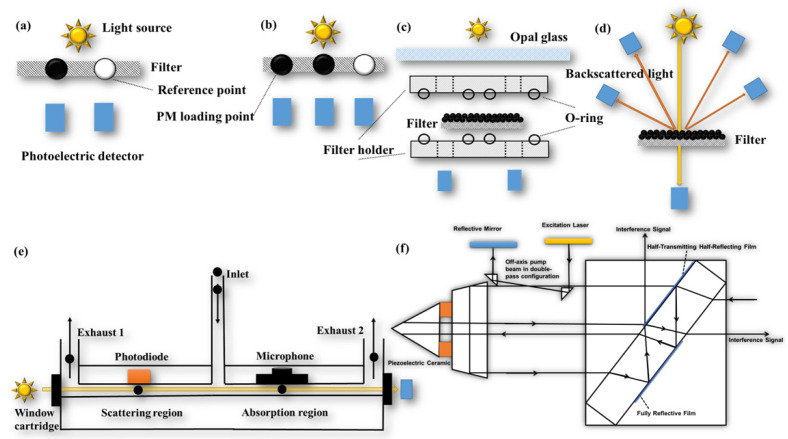
Schematic diagrams of several common instruments for measuring absorption coefficient: (**a**) single point AE; (**b**) dual-point AE; (**c**) PSAP; (**d**) MAAP; (**e**) PAX; (**f**) folded Jamin interferometer.

The filter-based online continuous measurement instrument allows for continuous sampling at multiple wavelengths with high temporal resolution (s). AE, PSAP, and the Multi-Angle Absorption Photometer (MAAP, company: http://www.thermo.com.cn/, accessed on 28 November 2023) are widely used optical online filter-based instruments. AE can be categorized into single-point method measurements (Figure 3a) and dual-point method measurements (Figure 3b) based on their measurement principles [131,134]. Single-point AE utilizes a filter strip with one PM loading point and one reference point, which is influenced by the PM loading effect. In contrast, dual-point AE has two PM loading points, one reference point, and two different flow rates to collect PM on the filter strip. This enables the simultaneous measurement of two different loading levels of ATN and provides a real-time loading effect compensation factor. The principle of PSAP (Figure 3c) involves the airflow passing through two filters in the instrument twice. By comparing the change in light transmittance before and after the airflow passes through the filter, the b_abs_ of the PM can be calculated [119,132]. MAAP (Figure 3d), on the other hand, addresses the issues of multiple scattering effects caused by the filter. It measures the backscattered light from multiple angles, which is used for scattering correction in the radiative transfer model, effectively eliminating interference from multiple scattering. Compared to AE and PSAP, MAAP significantly reduces the effects of loading and multiple scattering, with an uncertainty of only 12% [139,140].

### 3.2. In Situ Technique

The filter-based technique measures the b_abs_ of deposited PM, while the in situ technique measures the b_abs_ of PM in suspension. The in situ technique offers advantages such as real-time and continuous measurement, without interference from loading and filter scattering effects. However, it does not account for the bias of non-BC components on b_abs_ and is susceptible to interference from gas components and water vapor [129,141]. Common in situ techniques include using a Photoacoustic spectrometer (PAS, company: http://www.dropletmeasurement.com, accessed on 28 November 2023), Photo thermal interferometry (PTI, company: https://haze.si/, accessed on 28 November 2023) (Figure 3f), and the Differential Method.

The b_abs_ measurement accuracy of PAS is excellent, with an uncertainty of only 5% [113]. However, it has limitations in detecting larger particles (>2.5 μm) and is susceptible to RH interference. PAS is a spectroscopic technique based on the Photoacoustic effect. The sample is placed in a Photoacoustic cell and exposed to monochromatic light. The sample absorbs the light energy and converts it into thermal energy, causing the periodic warming of the sample and the surrounding medium according to the modulation frequency of the light. This leads to the generation of periodic pressure waves in the medium. A highly sensitive piezoelectric ceramic microphone detects these pressure waves and converts them into Photoacoustic signals. By tuning the wavelength of the incident light, a spectrum of wavelength-dependent Photoacoustic signals is obtained, representing the properties of the medium within the Photoacoustic cell. If a flowing absorption cell is used, online measurement of the aerosols becomes possible. Similar to PAS, PTI is also based on the photo-thermal effect, but it detects the temperature change in the air surrounding the aerosols using interferometry [126]. It relies on the refractive index change caused by the thermal effect, allowing for frequency modulation and effectively mitigating the influence of background noise. PTI exhibits advantages such as high sensitivity and fast response speed. Laser beam irradiation causes aerosols to absorb the laser energy, resulting in the warming of the surrounding air and the transfer of thermal energy. Interferometry is employed to measure the changes in the refractive index of the air, ultimately determining the aerosol b_abs_. Instruments like the Jamin interferometer and Mach–Zehnder interferometer utilize the PTI principle while compensating for mechanical vibrations [142]. The differential method is another approach used to indirectly obtain b_abs_ via calculating the difference between the extinction coefficient (b_ext_, Mm^−1^) and the scattering coefficient (b_sca_, Mm^−1^), i.e., b_abs_ = b_ext_ − b_sca_ [143]. The differential method can eliminate errors caused by light source fluctuations and mitigate the influence of ambient temperature and background factors [144]. It was developed earlier than the direct measurement method and allows for simultaneous measurement of extinction coefficients and scattering coefficients at multiple wavelengths or even continuous spectra. Combining spectroscopy with integrating sphere technology is a common practice. Nephelometers, which measure scattering coefficients, encounter truncation errors during the integration process and face challenges in backward scattering measurements. Additionally, the scattering effect of the ambient atmosphere outweighs the absorption effect, and water vapor can interfere with nephelometer measurements. Consequently, nephelometers perform better in artificial aerosol observations compared to real atmospheric conditions [129,145,146,147].

### 3.3. Comparison of b_abs_ Measurement Results of Different Optical Instruments

Despite numerous calibration efforts, the consistency of b_abs_ measurements from different optical devices in various observation environments still varies. The abundance of organic aerosols (OA) has an impact on the consistency of b_abs_ measurements between filter-based and in situ optical instruments, with the filter-based method being more affected. Zhang et al. [148] discovered that the detection capability of the PSAP varies with the variation in organic aerosol (OA) abundance in aerosols. It provides accurate measurements in areas with low OA abundance, but it overestimates b_abs_ by at least 50% in urban areas and by over 100% in heavily polluted areas. Lack et al. [149] compared ambient aerosol b_abs_ using PSAP and a Particle Absorption Spectrometer (PAS) at different OA abundances. Their results showed that the ratio of b_abs_ measured using PSAP to b_abs_ measured using PAS was 1.38 ± 0.01, with an R^2^ value of 0.78 throughout the study period. However, this ratio varied depending on the OA abundance. It was 1.12 when OA was low (<2.5 μg/m^3^) and 1.7 when OA was high (>12.5 μg/m^3^). This variation is primarily caused by the bias in PSAP measurements due to OA. The liquid organic particles, which are a major component of OA particle mixtures [150], are absorbed by the filter, altering the original physical structure of the fibers. As a result, scattering artifacts increase on the filter, surpassing the PSAP’s ability to correct for solid particles like ammonium sulfate and soot particles. Furthermore, the internal mixing of BC and OA on the filter enhances BC light absorption. Semi-volatile substances often contribute to negative PAS measurement biases (e.g., water vapor) [151]. Still, the RH consistently remained below 30% throughout the sampling process, making the consideration of PAS bias unnecessary. In a study by Tasoglou et al. [152], the b_abs_ of BB aerosols were measured using AE, MAAP, and Photoacoustic Extinctiometer (PAX). MAC values were obtained through combining rBC measurements from the soot particle–aerosol mass spectrometer (SP-AMS). The study revealed that OA abundance has an impact on the consistency of eBC results by influencing the actual MAC values of aerosols. When the OA abundance is low (OA/rBC < 0.1), the MAC values for the PAX_blue_ (405 nm), PAX_green_ (532 nm), AE (880 nm) and MAAP (670 nm) were 8.1, 6.5, 4.4, and 5.3 m^2^/g, respectively, resulting in better eBC agreement (slope range 0.85 to 0.98). However, with high OA abundance (0.1 < OA/rBC < 0.7), the MAC values for the PAX_blue,_ PAX_green,_ AE, and MAAP were 20.9, 15.2, 9.8, and 9 m^2^/g, respectively, leading to worse eBC agreement (slope range 0.74 to 1.46). This association can be attributed to the increased thickness of the BC coating as OA abundance rises, thereby enhancing BC absorption. Furthermore, light-absorbing OA, such as BrC, introduces a positive bias to optical instruments. Notably, AE consistently overestimates eBC compared to other optical devices, independent of OA abundance. This suggests that AE has the largest uncertainty among the optical instruments studied, possibly due to the choice of correction scheme for AE bias effects [153,154]. The C_ref_, which is the main source of uncertainty in the AE calibration scheme, cannot be considered constant due to its variation with filter material and PM type. When AE employs Teflon-coated glass fiber (TFE) filter tapes, the default C_ref_ is 1.57 [131]. Conversely, when using quartz filter tapes, the default C_ref_ is 2.14 [137]. However, the value of 2.14 is specific to the determination of fresh soot particles, diesel particles, and ammonium sulfate mixtures, and is not representative in general. Several subsequent studies have confirmed that the true C_ref_ for ambient aerosols within a fixed wavelength ranges from 3 to 8 [135,154]. Therefore, the use of AE in specific observational settings necessitates the meticulous calibration of the C_ref_ values, usually requiring in situ technical instruments (e.g., PAS, PAX) or more reliable filter-based instruments as benchmarks (e.g., MAAP). For instance, Davies et al. [127] discovered that the deviation between the Tricolor Absorption Photometer (TAP, company: https://www.brechtel.com/product/tricolor-absorption-photometer-tap/, accessed on 28 November 2023) and PAS in measuring BB aerosols was within ±30%. In light of this, Laing et al. [154] utilized b_abs, TAP_ as a benchmark to correct for b_abs, AE_. Through comparison, they found that the b_abs, AE_ obtained with a C_ref_ of 1.57 was 3.4 to 4 times larger than that of b_abs, TAP_. Subsequently, by applying further corrections, they derived a wavelength-independent correction factor (C_f_) of 4.35 to replace the C_ref_ when calculating b_abs, AE_.

Some specific observation environments, such as polar regions, exhibit low overall aerosol mass concentrations and minimal levels of light-absorbing components. These factors can potentially impact the precision of optical instruments. Asmi et al. [34] conducted optical property observations of ambient PM_10_ in Finland using both filter-based instruments [AE, PSAP, MAAP, and the Continuous Soot Monitoring System (COSMOS)] and difference-method instruments (EMS). Throughout the observation period, the prevailing atmospheric conditions consisted of Arctic clean transport air masses, resulting in deficient aerosol concentrations mainly dominated by scattering (single scattering albedo, SSA = 0.97). This atmospheric condition can lead to a substantial disparity between b_abs_, as measured with filter-based instruments, and b_abs_, as measured using the difference method. The range of b_abs_ using the filter-based (0–0.3 Mm^−1^), differs from the b_abs_ measured using the different method (0–3 Mm^−1^) by a factor of 10, primarily due to the significant error amplification effect exhibited by EMS when detecting aerosols dominated by scattered light. Although the detection errors of EMS in b_sca_ and b_ext_ are only 1–10%, the errors in b_abs_ resulting from phase subtraction range from 10–100% [143]. Each filter-based instrument displayed distinct measurement capabilities. Among them, b_abs_ and COSMOS yielded the lowest measurements due to the effective removal of scattered particles and BC coating through inlet heating. This reduction in bias was outlined by Kondo et al. [125], and additionally, the heating process minimized data fluctuations induced by RH. AE consistently exhibited the highest degree of b_abs_ overestimation, as indicated by studies conducted by Laing et al. [154] and Holder et al. [153], which can be attributed to the fact that the AE bias correction scheme does not account for Arctic-specific aerosol types. On the other hand, MAAP demonstrated relatively minimal measurement bias when utilizing the filter-based method [140]. Among the compared instruments, b_abs, AE33_ demonstrates the highest agreement with b_abs, MAAP_ in terms of the data trend (R^2^ = 0.87), followed by b_abs, COSMOS_ (R^2^ = 0.85), b_abs, PSAP_ (R^2^ = 0.78), and b_abs, AE31_, which exhibits the weakest correlation (R^2^ = 0.65). Regarding linear correlation, b_abs, AE31_ shows the closest resemblance to b_abs, MAAP_ (slope = 0.95), with b_abs, PSAP_ ranking second (slope = 0.93), followed by b_abs, COSMOS_ (slope = 0.68), and b_abs, AE33_, having the lowest similarity (slope = 0.62). The poor agreement between b_abs, PSAP_ and b_abs, MAAP_ stems from significant data noise introduced by PSAP during measurements in remote or highly polluted areas, resulting in subpar data quality without adequate sample pre-processing measures or appropriate calibration schemes [155]. The reason behind the weak agreement between b_abs, AE31_, and b_abs, MAAP_ lies in AE31’s tendency to have the highest amount of data noise among all the compared instruments. The largest deviation in value between b_abs, AE33_ and b_abs, MAAP_ occurs due to AE33’s inclination to overestimate b_abs_ during periods of low overall aerosol b_abs_ and underestimate b_abs_ when the overall b_abs_ is high. The observed significant deviation of b_abs, COSMOS_ from b_abs, MAAP_ can be attributed to the sample heating pretreatment utilized in COSMOS.

In conclusion, optical instruments, particularly filter-based instruments, commonly achieve a good consistency in detecting low OA content and weakly scattered PM, such as soot particles. However, the consistency deteriorates when detecting high OA content and PM dominated by scattering, primarily due to the measurement bias of b_abs_. The presence of non-BC components, including their optical properties and mixing morphology with BC, contributes significantly to the interference in quantifying the bias of BC. Additionally, the calibration scheme of b_abs_ in filter-based instruments is another critical factor affecting bias. Therefore, to obtain accurate eBC measurements using the optical method, the accuracy of b_abs_ and MAC must be ensured. For filter-based instruments, the accuracy of b_abs_ can be calibrated by utilizing benchmark instruments (e.g., PAX, PAS, MAAP) to develop a specific b_abs_ calibration scheme that precisely matches the aerosol type being measured [127,154]. It is essential to maintain a controlled external sampling environment, including minimizing water vapor, for the benchmark instrument. MAC can be obtained by combining the BC mass density measured with co-located instruments (e.g., carbon analyzer, single particle soot photometer (SP2), SP-AMS) with b_abs_ [34,152].

### 3.4. Sample Heating Pretreatment Method 

By taking advantage of BC’s high refractoriness, heating the inlet can partially reduce the bias caused by non-BC components (such as OA and secondary inorganic salts) in PM, thus enhancing the consistency of measurement results among optical instruments [156]. To eliminate the bias resulting from OA, Kanaya et al. [157] employed a 400 °C heating device for the PSAP inlet. They discovered that the regression line slope (eBC_heating_/eBC_no-heating_) was 0.70 ± 0.01 (R^2^ = 0.92), indicating that utilizing the heated inlet tube resulted in eBC concentration readings that were 30% lower compared to using the unheated tube when employing an identical MAC (10 m^2^/g, 565 nm). This observation suggests that, on average, the MAC_no-heating_ is 30% higher than that for MAC_heating_. To alleviate the impact of VOCs and scattering particles on b_abs_ measurements, Kondo et al. [125] employed air intake heating up to 400 °C for a duration of 0.3 s. Notably, significant changes were observed in MAC values at 565 nm for PM_2.5_ (or PM_1_) samples collected at five locations, ranging from 1.17 to 1.67 for MAC_no-heating_/MAC_heating_. These changes can be attributed to the implemented heating measures, which effectively removed most of the sulfate, nitrate, ammonium, and organic matter present in the samples. The removal of these substances contributes to a reduction in the positive deviation of b_abs_ [125]. However, it appears that the heating measures have minimal impact on BC with a higher degree of aging. When oleic acid serves as the BC coating, the MAC only experiences a slight increase when D_p_/D_BC_ < 1.5 (particle diameter/diameter of BC, μm). Nevertheless, in cases where 2.0 < D_p_/D_BC_ < 2.5, the MAC does not undergo a significant increase, instead, it decreases. This phenomenon can be explained by considering the larger D_p_/D_BC_ ratio, which results in a shallower penetration of coated BC particles into filter fibers. Consequently, this compensates for the amplification of light absorption caused by internally mixed BC. The phenomenon described above was also observed in a study by Knox et al. [124], where they conducted an experiment involving the heating of ambient PM_2.5_ samples to 340 °C (0.56 s). They found that this heating process caused more than 80% of the non-BC fraction to vaporize, resulting in a change in the MAC of BC. However, the extent of this change varied based on the degree of aging of the BC. The MAC at 760 nm of fresh BC decreased from 9.3 ± 1.8 m^2^/g to 7.7 ± 2.2 m^2^/g, while that of semi-aged BC decreased from 9.9 ± 2.0 m^2^/g to 6.9 ± 2.2 m^2^/g. On the other hand, there was no statistically significant change in the MAC of fully aged BC. These observations indicate that heating methods effectively eliminate volatile components in incompletely aged BC but have limited impact on fully aged BC due to the thicker initial coating. The loss of coating material caused by heating is insufficient to result in significant changes in MAC. In other words, MAC varies depending on the thickness of the coating, as long as it remains below a certain threshold. Therefore, the use of sample heating pretreatment technique aims to improve the consistency of b_abs_ measurements obtained from different optical instruments, particularly for samples with lower levels of aging.

## 4. Refractory Black Carbon

The LII technique [30,37,158] (Table 8), distinct from traditional thermal and optical methods, emerged in the 1970s to measure soot particles emitted from combustion. It has since undergone rapid development, with notable commercial instruments being the SP2, SP2-XR (company: https://www.dropletmeasurement.com/product/single-particle-soot-photometer-extended-range/, accessed on 28 November 2023), and SP-AMS [156,159,160]. The fundamental principle behind LII is to exploit the fire-resistant characteristics of BC and employ a high-energy pulsed laser beam to irradiate an aerosol containing BC. This irradiation rapidly raises the temperature of BC from the flame temperature (~2000 K) to the vaporization temperature (~4000 K). As a result, incandescent light emission occurs from BC, serving as an indicator to assess the quality of rBC based on the intensity of the incandescent signal. The detection signal of non-BC components is caused by scattering, enabling inference of the particle size distribution of rBC core and coatings, as well as their mixing state with rBC [30]. And neither TOA nor optical methods can provide information such as that from LII outlined above. A big difference between them is that LII detects resolved particles, while the TOA and optical methods detect particles. The traditional tool for observing the mixed state of BC is electron microscopy (including transmission and scanning electron microscopy), which is derived from direct images observation rather than parametric quantification [161,162,163]. Therefore, LII is an ideal instrument for studying the physicochemical properties and aging degree of individual BC particles; however, it also has bias effect when quantifying rBC mass concentration. This is because, in order to ensure complete evaporation of all sample components, only a fraction of the particles within the detection range (PM_1_) can enter the detection system [135]. As a result, there is an underestimation of the rBC mass [31]. For instance, Wang et al. [164] demonstrated that the LII instrument significantly underestimates (>50%) the mass of larger-sized BC particles (>1 μm) in PM_10_. Apart from the limitations in particle size detection imposed by technical constraints, uncertainties also arise from the selection of calibration materials and the mass loss correction scheme employed in the LII technique. In addition, for SP-AMS, the reasonable selection of collection efficiency is also a key factor to determine the accuracy of rBC quantification. Nonetheless, it should be acknowledged that the measurement of rBC mass using LII shows little dependence on the physical and chemical properties of the aerosol, such as its chemical composition, mixing state, degree of aging, etc. Consequently, the detection limit of LII primarily stems from the instrument itself, rather than being heavily influenced by aerosol properties as with TOA and optical methods.

### 4.1. Single Particle Soot Photometer

**Figure 4:**
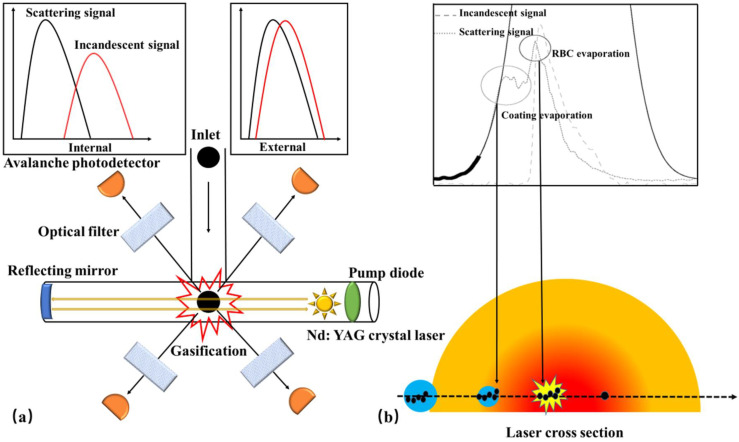
Schematic diagrams of refractory black carbon (rBC) detection instruments: (**a**) SP2; (**b**) SP-AMS [37].

The SP2 instrument (Figure 4a) offers the advantage of being sensitive and independent of the mixing state when quantifying rBC. It provides real-time information on the mass concentration, size distribution, and coating thickness of individual BC particles. Moreover, it is widely recognized as the most commonly employed device for measuring BC mass distribution as a function of size and mixing state [165,166,167,168,169]. When introduced into the SP2, the sample intersects with a continuous high-intensity intracavity Nd: YAG laser beam (λ = 1064 nm) that operates at 1 MW/cm^2^. As the rBC absorbs the optical energy, it rapidly vaporizes, resulting in an incandescent signal. The rBC mass is determined by establishing a linear relationship between the incandescent signal and rBC mass. Simultaneously, the scattering signal allows for the inference of both the coating thickness and the mixing state of individual BC particles.

The uncertainty of SP2 arises from the selection of calibration materials and the rBC underestimation resulting from particle size detection range. Variations in the chemical microstructure of BC emitted from different sources imply the need for corresponding calibration materials for different BC types. However, it is impractical to develop complementary calibration materials for all BC types [170]. Only a few studies have manufactured calibration materials using study samples, such as Kondo et al. [156], who extracted particles that survived evaporation after passing through an inlet heated to 400 °C as SP2 calibration material. Nonetheless, most studies have employed commercially available calibration materials, including Fullerene Soot (similar to diesel emission particles and Tokyo ambient samples) and Aquadag, which both exhibit excellent chemical stability and mass coverage. Nevertheless, utilizing different calibration materials can introduce bias into rBC quantification. For instance, in some BC with particle sizes smaller than 350 nm, Aquadag exhibits an incandescent peak amplitude that is 40% larger than that of Fullerene Soot [165,171,172]. Additionally, Miyakawa et al. [173] discovered that calibration with Aquadag introduces a positive bias of approximately 20% in rBC for ambient samples. The particle size of SP2 depends on technical limitations and parameter settings, with optimal detection performance reaching 0.12 fg/particle [171,174]. This corresponds to a lower detection limit (LDL) of 50 nm (mass equivalent diameter) when detecting solid BC with a bulk density of 1800 kg/m^3^ [172]. If the optimal settings are not used, the particle size can be increased to 80 nm (0.48 fg/particle). The upper detection limit (UDL) of particle size, typically ranging from 500 nm to 1 μm, is determined by the detector performance [31]. Therefore, mass loss in rBC occurs beyond the LDL and UDL, requiring further correction for accurate data. The extent of mass loss depends on the air quality. In remote areas influenced by long-range air mass transport and aging processes, the SP2 instrument can only detect approximately 50% of the rBC. Conversely, in urban areas with a higher abundance of freshly emitted small-sized BC particles, a correction of only 25% for rBC mass concentration is necessary [29,175]. Certain chemical components, such as metal oxides and volcanic ash, can affect the quantification of rBC. These components exhibit strong fire resistance and light absorption at 1064 nm, producing incandescent signals similar to rBC [176,177]. Additionally, Sedlacek et al. [11] found that some organic components undergo pyrolysis at high laser power, leading to an overestimation of rBC. Therefore, comparing quantitative results obtained using the SP2 instrument among different researchers is complex due to variations in instrument performance, calibration materials, atmospheric conditions, and mass loss correction schemes. These factors are usually not directly compared [167].

### 4.2. Soot Particle–Aerosol Mass Spectrometer

Although SP2 provides rich physical information about BC, it cannot detect the chemical composition of the coating. High resolution-particle time-of-flight aerosol mass spectrometer (HR-ToF-AMS) enables the real-time measurement of the chemical composition of submicron non-refractory particles (NR-PM) [178,179], including organic matter, sulfates, nitrates, ammonium salts, and chloride, among others. NR-PM is evaporated in a tungsten evaporator (600 °C), ionized with a 70 eV electron beam, and detected in a V-mode high-resolution mass spectrometer (In V-mode, ions follow a traditional reflectron path, the resolution of which is 2500) [178]. However, HR-ToF-AMS is unable to detect refractory particles (R-PM). To address this limitation, SP-AMS (Figure 4b) combines HR-ToF-AMS and SP2, making it the only instrument capable of online detection of BC’s mass, particle size, and chemical composition. It plays an indispensable role in characterizing various aspects of BC, such as its source, mixing state, atmospheric life, and aging mechanism [180]. Onasch et al. [37] incorporated the design of SP2 and integrated an intracavity Nd: YAG laser evaporator into HR-ToF-AMS, facilitating the evaporation of R-PM that goes undetected by the instrument. By utilizing ionization detection, HR-ToF-AMS effectively characterizes the R-PM [37]. The two evaporators of SP-AMS can operate independently to characterize pure organic particles and BC-containing particles, respectively. When the laser evaporator is turned off, SP-AMS functions in the same way as HR-ToF-AMS for evaporating pure organic particles (TV mode, tungsten vaporizer). Conversely, when the laser evaporator is turned on, SP-AMS is used to measure BC-containing particles (LV mode, laser vaporizer), albeit generating some organic particle signals due to the evaporation of BC-organic mixed particles, which cannot be detected in LV mode [37].

The uncertainty of SP-AMS primarily lies in the collection efficiency (CE), which is used to describe the effective detection of the mass of particles after undergoing mass loss through a sampling tube, time-of-flight chamber, and evaporator. The CE of the tungsten evaporator is mainly controlled by the particle bounce effect, while the CE of the laser evaporator is primarily affected by the divergence of non-spherical irregular particles [181,182,183]. Particle beams characterized by more compact shapes and uniform mass sizes tend to exhibit lower susceptibility to particle beam divergence compared to beams with diverse shapes, masses, and sizes. They maintain higher concentration levels as they traverse the laser beam, minimizing the loss of particles due to flight divergence. Consequently, this leads to a higher CE value [37]. A study conducted using a beam width probe examined the CE of different forms of BC (bare and internally mixed). The findings revealed that the morphology of BC influences the degree of overlap between the particle beam and laser beam, subsequently affecting the CE. This implies that the rBC measured by SP-AMS is influenced not only by the CE but also by the shape of BC and the particle beamwidth. Unfortunately, these factors are beyond control and inevitably lead to an underestimation of the rBC mass [152,184,185]. Additionally, the coating composition, geometric shape, and phase distribution of BC can also impact the CE of SP-AMS [181,185,186]. Therefore, the accuracy of the CE plays a crucial role in determining the precision of SP-AMS in measuring rBC.

### 4.3. Correction Scheme for Refractory Black Carbon Mass Loss

The rBC mass loss correction scheme used by SP2 is relatively simple. Two standard methods are commonly employed: the extrapolation and fitting methods [168,187]. Both methods utilize the unimodal lognormal function formula to fit the measured values of rBC. This approach is justified because the mass distribution of BC across different particle sizes closely follows a lognormal distribution. The extrapolation method corrects SP2 rBC measurements by extrapolating the measured size distribution below and/or above the SP2 detection limits. By modifying the measured mass, the true mass of the rBC is determined. On the other hand, the fitting method assumes that the true BC mass size distribution in the submicron size range precisely conforms to a lognormal function. Under this assumption, the corrected rBC mass is obtained by integrating the mass of a lognormal fit to the measured rBC mass size distribution, which includes adjustments for contributions below the LDL and above the UDL. The main differences between the two methods lie in the fitting residual and the estimation of mass loss surpassing the UDL. However, the variation in correction effect is consistently insignificant. For instance, Pileci et al. [31] reported that the average difference between the two methods after correcting for Melpitz winter and ambient summer samples was merely 3%.

The CE of SP-AMS is affected by multiple factors, resulting in varying degrees of deviation in uncorrected rBC measurements [152,181,185,188,189]. Consequently, researchers have employed various CE correction schemes to improve the consistency between rBC_SP-AMS_ and other measurement instruments. Some scholars have used co-located quantitative instruments for BC to calibrate SP-AMS and derive fixed CE values. For example, Fortner et al. [189] calibrated the CE using a MAAP, establishing a CE value of 0.15 based on the linear relationship between elemental carbon measured using MAAP (eBC_MAAP_) and rBC_SP-AMS_ in flame particles. Similarly, Dallmann et al. [188] determined a CE value of 0.27 by comparing the linear relationship between eBC_MAAP_ and rBC_SP-AMS_ in vehicle exhaust studies. Other researchers have adopted more direct methods to obtain CE values. Tasoglou et al. [152] used a beamwidth probe to measure the beamwidth of BB particles, resulting in a CE value of 0.35. Additionally, an empirical value of CE = 0.5 is commonly applied to most environmental samples, leading many researchers to directly adopt this value during environmental observations [178,182,190]. However, the application of a CE value of 0.5 does not universally apply to all ambient samples. For instance, Middlebrook et al. [186] discovered that utilizing a default CE of 0.5 for various campaigns (such as those involving acidic sulfate particles, an aerosol with a high mass fraction of ammonium nitrate, and an aerosol primarily composed of BB emissions) yielded an 81–90% agreement between the AMS speciated and total mass concentrations, in comparison with fine particle volume or particle-into-liquid sampler (PILS) measurements, within experimental uncertainties. However, there were positive biases when compared to a random error curve. To address this, they developed an algorithm to estimate CE based on the aerosol’s chemical composition and the sampling line RH. By incorporating composition-dependent CE values, which increased the CE for the aforementioned aerosol types, the data points falling within measurement uncertainties rose to over 92%, while the mass concentrations decreased by approximately 5–15% on average. In addition to the chemical composition, coating thickness is a crucial factor influencing the bias of CE. In their study on CE correction in SP-AMS, Collier et al. [184] observed that the relationship between rBC_SP2_ and rBC_SP-AMS_ (assuming CE = 1) exhibited a slope of 0.37 and a Pearson’s r correlation of 0.78. Conversely, after removing the coating material using thermodenuder conditions, the scatter plot showed an improved correlation (r = 0.82) but a reduced slope (0.24). The stronger correlations in the thermodenuder data are likely attributed to the evaporation of most rBC coatings, resulting in a more consistent CE value. However, when the coating thickness exceeds a certain threshold, particularly at R_coat_/rBC > 2.5 (where R_coat_/rBC represents the mass ratio of total non-refractory material to rBC, used to quantify CE changes), the apparent CE (rBC_SP-AMS_/rBC_SP2_) is no longer constant. In essence, the deviation in CE for SP-AMS measurements is influenced by various factors, such as coating morphology and particle chemical composition [181,185,186,191]. Therefore, it is essential to comprehensively consider these factors in order to achieve more accurate measurements of rBC_SP-AMS_.

## 5. Inter-Comparison of Black Carbon Quantification between Techniques

The discrepancy in EC measurement primarily arises from the selection of thermo-optical protocols and the interference caused by non-BC chemical components. This bias in EC quantification, particularly due to PC, can be effectively mitigated through solvent extraction. The primary uncertainty of eBC measurement is the aging of BC, which primarily manifests as a deviation in the b_abs_ measurement caused by changes in the original MAC. Additionally, filter-based instruments exhibit strong sensitivity towards the choice of b_abs_ correction scheme, necessitating the use of a scheme that precisely corresponds to the PM type. To enhance the consistency of optical instrument detection and minimize the impact of BC aging on b_abs_ measurement, heating the sample can be employed. The quantitative technique for rBC offers the highest sensitivity and least uncertainty at present. However, its particle size detection range (1 μm) limits its suitability for observing BC mass above the submicron scale. Furthermore, the PM type is susceptible to correction materials (e.g., SP2) and mass loss correction schemes (e.g., SP-AMS). Therefore, it is crucial to accurately identify the PM type and subsequently select appropriate correction materials and mass loss correction schemes. Subsequently, we will conduct an inter-comparison of the three aforementioned quantitative techniques, combined with previous studies, to characterize the variations in their response to different observable conditions and analyze the underlying reasons.

### 5.1. Refractory Black Carbon vs. Elemental Carbon

Both TOA and LII technologies utilize the fire resistance properties of BC to quantify its mass. Consequently, in most instances, there is a good data consistency between rBC and EC. However, it should be noted that rBC measurements often yield smaller values compared to EC. This discrepancy primarily arises from the fact that EC has a maximum oxidation combustion temperature below 1000 °C. Thus, it is possible that certain substances with superior heat resistance could positively bias the quantitative measurement of EC. On the other hand, rBC is a highly refractory component measured at 3600 °C, which means that the influence of OC on the results is negligible. Furthermore, due to the extended heating time, TOA tends to generate more PC than LII. As a result, the deviation in EC measurements is greater compared to rBC [192]. For particulate matter containing minor non-BC components, EC and rBC present a better data closure. For instance, Laborde et al. [165] demonstrated that the consistency between rBC_SP2_ and EC in laboratory-generated Combustion Aerosol Standard (CAST) is high, with the mass concentration of EC falling within ±15% of rBC. This is attributed to the reduced presence of OC impurities (OC/TC = 60%) in CAST, resulting in a significant decrease in EC deviation caused by PC. Additionally, the particle size distribution of CAST aligns closely with the detection range of SP2, indicating minimal uncertainty associated with rBC measurements. One important reason is the limitations of the LII instrument in detecting particle sizes, which results in quality loss. Miyakawa et al. [173] conducted a study on industrial soot using SP2 and TOA in Yokosuka, Japan. This soot aerosol, emitted from combustion sources, is primarily unrelated to BC aging. The results indicated that EC was 44% greater than rBC, showing a strong correlation coefficient (R^2^ = 0.98). The underestimation of rBC is attributed to a 15% mass loss caused by limitations in detecting particle sizes with the SP2. Additionally, the overestimation of EC is due to a positive deviation resulting from OC pyrolysis during sampling. In certain cases, such as marine diesel engine exhaust particles, the proportion of chemical components may vary with engine operation. Corbin et al. [193] investigated rBC and EC in marine diesel engine exhaust using SP2 and TOA. They observed that the relationship between the two variables relied heavily on the presence of light-absorbing organic matter, specifically tar. When BC dominates as the light-absorbing component, the AAE of PM is at its lowest (~1.0), and EC/rBC = 1.0. Conversely, when organic matter predominantly absorbs light, the AAE increases (close to 2.0), and rBC/EC approaches 0. This deviation occurs because MAC_tar_ < MAC_EC_, leading to an overestimation of EC. Even if the tar has a higher gasification temperature, SP2 can vaporize it at the initial stage, eliminating the interference of tar on rBC quantification.

Variations in aerosol types are observed in ambient samples across different regions, which are primarily manifested through differences in the chemical composition of non-BC impurities and variations in BC particle size distribution. Consequently, unifying the size relationship between EC and rBC appears challenging. For instance, Zhang et al. [194] reported that rBC_SP2_ levels were 30% lower than EC levels in ambient PM_2.5_ samples from Fresno, California. This discrepancy mainly stems from the underestimation of rBC due to systematic mass loss, improper calibration material selection (Fullerene soot), and uncertainty in the lognormal fitting correction scheme. Sharma et al. [192] explored ambient PM_1_ levels in the Canadian Alert and discovered that EC exceeds rBC by a factor of 3.1. The underestimation of rBC primarily stems from the mass loss calibration scheme, while the parameterized particle density estimation method may deviate from the actual particle density [195]. The strong correlation between PC and EC/rBC unequivocally demonstrates that the overestimation of EC originates from the pyrolysis of OC. Pileci et al. [31] conducted an observational campaign employing SP2 and TOA at various atmospheric background sites across Europe (Palaiseau, Bologna, Cabauw, Melpitz). Overall, a minor systematic bias in rBC and EC exists, with rBC/EC = 0.92. However, the variation in rBC/EC is larger for individual observation campaigns, ranging from 0.53 to 1.29. The rBC shows an underestimation primarily due to mass loss caused by limitations in the SP2 particle size detection range. When the particle mixing state is external (internal), the SP2 UDL ranges from 625–970 nm (1140–1660 nm). Consequently, a portion of the rBC mass between PM_1_ and PM_2.5_ remains undetected. This explains the occurrence of rBC/EC < 1.0 in single observation campaigns, such as rBC/EC_Cabauw_ = 0.53, rBC/EC_Melpitz, summer_ = 0.97, and rBC/EC_Bologna_ = 0.65. However, the authors fail to provide a reasonable explanation for the phenomenon of rBC/EC > 1.0, like rBC/EC_Melpitz, winter_ = 1.29. Notably, the Melpitz winter samples exhibit the highest EC loadings and AAE among all the samples, suggesting a greater abundance of OC (especially BrC) in those samples. Previous studies have indicated that this observational activity tends to underestimate EC to a greater extent than rBC. The primary reason for the EC underestimation is the transmittance saturation effect triggered by high EC loading, which hinders the establishment of a normal linear relationship between EC and transmittance signals [196]. Secondly, the combined effect of particle composition and temperature program leads to MAC_PC_ > MAC_EC_, which further delays the appearance of OC-EC splitting points and triggers an underestimation of EC [62,193]. Considering the interference of non-BC components with high heat resistance and strong light-absorption in PM, Corbin and Gysel-Beer [193] proposed a detailed division for the traditional light-absorbing carbonaceous components: soot BC, char BC, tar BrC, and soluble BrC, and outlined their respective physicochemical properties. This comprehensive classification provides a novel insight into the light-absorbing components in PM and offers a new approach to quantitatively differentiate between TOA and LII. For instance, tar BrC represents an amorphous carbonaceous form that exhibits high refractoriness, causing a positive bias in EC measurements without significant interference in rBC quantification.

### 5.2. Equivalent Black Carbon vs. Elemental Carbon

The optical method indirectly determines the mass concentration of BC by exploiting its light absorption properties. The key uncertainty associated with this approach lies in the modification of MAC induced by non-BC components. However, to ascertain whether the modified MAC is greater or smaller than the manufacturer’s default value, it is necessary to compare it with measurements obtained from co-located instruments. Consequently, both overestimation and underestimation of eBC quantitative deviation are possible outcomes. In most cases, the presence of non-BC components tends to enhance the light absorption of BC, resulting in an overestimation of eBC. On the other hand, the TOA method employs the high heat resistance of EC to quantify its mass concentration. The primary source of uncertainty in this process is the pyrolysis of OC. However, determining whether the presence of PC leads to an overestimation or underestimation of EC quantification bias using conventional single-wavelength thermo-optical instruments poses a challenge. Judging the thermal spectra before and after solvent extraction is essential in this regard. Therefore, the relationship between the magnitudes of eBC and EC is not absolute and must be evaluated within the specific atmospheric observation conditions.

Ambient samples exhibit significant variations in composition, which are influenced by geographical and seasonal factors. For instance, Sharma et al. [197] conducted a study on ambient samples collected from urban and remote areas in Canada, utilizing AE, PSAP, and TOA techniques. They observed substantial disparities in eBC/EC ratios due to instrumental, geographic, and seasonal differences. These variations originate from the diverse aerosol compositions and morphologies, leading to fluctuations in aerosol MAC values (ranging from 6.4 to 28.3 m^2^/g at 880 nm). Consequently, this introduces biases into the quantification of eBC. The distinguishing factor among optical instruments primarily lies in the detection wavelength (λ). At λ_AE_ = 880 nm, the default correction factor is MAC_AE_ = 19 m^2^/g, resulting in an eBC/EC_AE_ variation range of 0.31–5. Meanwhile, at λ_PSAP_ = 565 nm, the correction factor is MAC_PSAP_ = 10 m^2^/g, yielding an eBC/EC_PSAP_ variation range of 0.31–1.2. In this analysis, we will mainly focus on the AE measurements. Regarding geographical variations, apart from Alert (eBC/EC_Alert, winter_ = 1, eBC/EC_Alert, summer_ = 1.5), eBC was consistently underestimated compared to EC by as much as 76% in other regions.

The uniqueness of the eBC/EC ratio in Alert lies in its remote location, which makes it more susceptible to long-range transportation of aged air masses. As a result, BC with an internal mixing state predominates in Alert. This leads to a noticeable enhancement effect on light absorption, with MAC_Alert_ being approximately four times higher than at other sites. Conversely, in urban areas, the majority of aerosol types consist of externally mixed BC being emitted freshly. The MAC_true_ value is lower than the default MAC value because externally mixed BC does not exhibit the same light absorption enhancement effect. The seasonal disparity in Alert is particularly pronounced due to the high wind speeds in summer, resulting in roughly twice as much soil dust content in the summer aerosols compared to winter [198]. Specifically, the MAC_Alert_ (880 nm) in summer is 28.3 m^2^/g, while in winter it is 18.8 m^2^/g. Hence, maintaining consistency between EC and eBC relies on accurately determining the MAC value, which is closely associated with the aerosol types. Relying solely on manufacturer default values often leads to significant deviations. Kanaya et al. [157] found higher eBC levels than EC in ambient samples from Mount Tai in China, and a positive correlation between eBC/EC and OC/EC. This can be attributed to two reasons: First, the strong correlation between higher eBC/EC and lower NO_x_/NO_y_ indicates that aged BC is coated with transparent materials, leading to a lens effect that overestimates eBC levels. Second, the increase in OC content results in increased production of PC during the aging process. The presence of PC delays the splitting point of OC-EC, leading to an underestimation of EC levels.

Ahmed et al. [199] investigated the eBC and EC of four sampling sites using the Magee Scientific OT21 Transmissometer (OT21) and TOA. In general, there is excellent consistency between the two methods with an eBC/EC ratio of 0.91 and an R^2^ value of 0.84. However, significant regional differences in individual observations exist. The range of eBC/EC varies from 0.75 to 1.02 across different sampling sites, which can be mainly attributed to variations in aerosol loading, chemical composition, and mixing state. For instance, in Albany, where the sampling site is near a street and exposed to traffic emissions [200], the eBC/EC ratio is 1.02. This higher value is likely due to a higher MAC for BC than the default value provided by the manufacturer. On the other hand, in Antalya, the eBC is slightly overestimated (eBC/EC_Antalya_ = 1.02) due to the influence of the long-distance transport of aging air masses containing lots of iron oxides (hematite and goethite) from Africa which enhance the light absorption of BC [199]. At Whiteface Mountain, which is also affected by long-distance transport of aged air masses, BC is mixed with a significant amount of sulfate. The increase in the radius of sulfate coating accompanies a decrease in the MAC of BC (due to light saturation effect), resulting in the underestimation of eBC. This underestimation of eBC due to the light saturation effect is also evident in the observations conducted in Mayville (eBC/EC_Whiteface Mountain_ = 0.92, eBC/EC_Mayville_ = 0.75). In a study by Sharma et al. [192] on ambient samples from Alert, Canada, the EC and eBC were analyzed using TOA, AE, and PSAP. Throughout the entire observation period, eBC/EC = 0.87. The overestimation of EC arises from the pyrolysis of OC, while eBC exhibits significant seasonal variations. During cold months, influenced by long-distance transport and Arctic haze [201], the mass concentration of BC and non-BC light-absorbing constituents increases, leading to an enhancement in BC’s light absorption. Moreover, the deposition of high-concentration PM on filters results in stronger multiple scattering and PM loading effects, thereby causing an overestimation of eBC (eBC/EC_fall_ = 1.0). In warmer months in Alert, when the overall BC concentrations are lower than cold mouths, the extinction effect of PM is mainly due to scattering [34,192]. If the manufacturer’s default MAC is used continuously, it would result in the underestimation of eBC (eBC/EC_summer_ = 0.24).

Karanasiou et al. [202] employed MAAP, AE, and TOA to investigate the eBC and EC levels in PM_2.5_ at urban and regional sites in Barcelona, Spain. Generally, the eBC/EC ratio was found to be 1.2 with an R^2^ value of 0.79. Notably, there were distinct regional disparities observed within a single monitoring campaign. At the urban site, the ratios eBC_AE_/EC and eBC_MAAP_/EC both equaled 1.2, whereas at the regional site, these ratios were 1.9 and 1.7, respectively. These variations primarily stem from the non-BC constituents and the mixing state of BC. The aerosols at the regional site are influenced by the long-range transport of aged air masses. Consequently, the concentrations of non-absorbing or less-absorbing particles (such as sulphates, organic matter) in PM are significantly higher in comparison to urban areas. These substances are internally mixed with BC, resulting in a lens effect that enhances BC’s MAC. Conversely, most of the BC particles in urban areas are freshly emitted locally, and thus, the light absorption enhancement effect is not prominent. Consequently, the eBC/EC ratio for urban areas (eBC/EC_urban_) is lower than that for regional areas (eBC/EC_region_). In a study by Liu et al. [28] in Beijing, China, AE and TOA were utilized to measure eBC and EC in atmospheric aerosols. The results demonstrated a strong correlation between eBC and EC throughout the entire observation period (r = 0.90), indicating that both methods can simultaneously reflect the physicochemical properties of BC. The eBC values were consistently larger than the EC values, accounting for approximately 90% of the sampling time, with an average difference of 1.21 μg/m^3^ (33% of the mean eBC value). The eBC/EC exhibits distinct seasonal patterns, with eBC/EC_spring_ (1.67) > eBC/EC_autumn_ (1.15) > eBC/EC_winter_ (1.09) > eBC/EC_summer_ (0.91), which can be attributed to seasonal-specific pollutant sources altering the chemical composition of PM and subsequently influencing the MAC of BC. For instance, the prevalence of sandstorms during spring, intense biomass burning in autumn, and heightened coal combustion for heating in winter all contribute to an increase in light absorption by BC. Furthermore, a direct correlation between coating thickness and the magnitude of eBC/EC exists. As the coating thickness (primarily composed of SOC and nitrate) increases, the aging of BC intensifies, leading to subsequent elevations in MAC and an overestimation of eBC, ultimately resulting in higher eBC/EC values [28].

The aforementioned research indicates that the disparity between eBC and EC is directly related to the presence of non-BC constituents, such as organic matter and secondary inorganic salts, in aerosols. This discrepancy becomes more pronounced during periods of intense pollution. For instance, Jeong et al. [200] conducted a study on smoke particles before and after a forest fire, utilizing the TOA and the AE. They observed a considerable increase in OC, EC, and eBC by factors of seven, nine, and four, respectively, following the forest fire. Moreover, the eBC/EC ratio exhibited a sharp decline from 3.61 to 0.35 post fire. This change was attributed to a significant rise in atmospheric OA and moisture induced by wood combustion, consequently altering the MAC of smoke particles. Patterson and McMahon [203] documented that the actual MAC at 632.8 nm of smoke from fires ranged from 0.04 to 1.00 m^2^/g, which is notably smaller than the value of 16.6 m^2^/g at 680 nm recommended by the manufacturer used by Jeong et al. [200]. As a result, an underestimation of eBC occurred. Reisinger et al. [90] investigated the discrepancy between eBC and EC in ambient samples collected in Vienna, Europe, along with the impact of BrC on the comparability of eBC and EC measurements. Their findings revealed that eBC measured using MAAP exceeded EC_TOT-NIOSH_ values by 172.79%. The underestimation of EC_TOT-NIOSH_ arises from excessive T_peak_, whereas the overestimation of eBC results from the presence of non-BC light-absorbing constituents, such as BrC. This is most evident in the aerosols affected by biomass fuel heating in winter. Specifically, the difference between eBC and EC increases as the contribution of BrC increases. Zhi et al. [204] investigated the EC and eBC levels during the transition from clean days to heavy haze in Shanghai, using TOA and AE. They found that the eBC/EC ratio varied under different weather conditions. During clean weather, the eBC/EC ratio was 0.92, but it increased to 1.88 during periods of heavy haze. This increase can primarily be attributed to elevated PM concentrations, which enhance the internal mixing of BC with other species such as sulfates, nitrates, ammonium salts, and SOC. Consequently, this overestimates the eBC values. Based on the aforementioned studies, eBC and EC cannot be used interchangeably, particularly in situations where BC has undergone significant aging and pollution sources are diverse. While there is no fixed size relationship between eBC and EC, the general trend is that eBC/EC > 1.0 in most cases [205,206,207,208]. The degree of difference between the two parameters mainly depends on non-BC chemical composition, BC mixing state, and BC aging degree.

### 5.3. Equivalent Black Carbon vs. Refractory Black Carbon

The quantification of eBC in this context is prone to positive bias due to variations in BC mixing state and coating. To ensure accurate quantification, the rBC is heated to approximately 3600 °C prior to analysis, effectively preventing any interference from the coating. However, limitations in the detection range for particle size often result in the underestimation of small and large particle sizes beyond this range, leading to negative deviation in quantification. This conclusion has been supported by several researchers, such as Slowik et al. [209], who investigated soot particles (with a mobility diameter range of 150–460 nm) generated by a flame generator using Aerosol Mass Spectrometer-Scanning Mobility Particle Sizer (AMS-SMPS), SP2, MAAP, and PAS. Their findings indicate that, while optical instruments are significantly impacted by organic coatings like oleic acid and phenanthrene, LII instruments show negligible influence. The consistency among uncoated particles measured using AMS-SMPS, SP2, and PAS instruments falls within a range of less than 15%, whereas MAAP results in approximately 50% higher yields. Thin organic coatings (~10 nm) and thicker oleic acid coatings (~50 nm) exhibit minimal impact on instrument readings. However, thicker coatings (~60 nm) cause a 20% increase in MAAP readings and a 65% increase in PAS readings, while not affecting AMS-SMPS and SP2 readings. These disparities stem from differences in the refractive index of the organic coating.

Buffaloe et al. [210] employed four instruments, namely PAS, PSAP, SP2, and SP-AMS, to investigate the presence of BC in plumes emitted by 70 ships in California. The findings revealed a strong concurrence between eBC_PAS_, eBC_PSAP_, and rBC_SP-AMS_ readings. However, rBC_SP2_ measurements were consistently approximately half of the values obtained from the other three instruments. This disparity can be attributed to the limited particle size detection range (the calibration material was size-selected fullerene soot particles), as the detected particle size range is concentrated within the range of 60 to 300 nm. Holder et al. [211] assessed BC levels in aerosols emitted from road traffic using the SP2, PASS, and Aethalometer (AE, company: https://www.aerosolmageesci.com/, accessed on 28 November 2023) techniques, respectively. The results indicate that all instruments demonstrate good consistency (R^2^ = 0.80–0.89), albeit with a slope range of 0.52–1.03. Among them, the optical instruments PASS and AE exhibit the highest level of consistency, with a slope of 1.02 and R^2^ = 0.82. The rBC shows the lowest levels, being 40–54% smaller compared to eBC. The underestimation of rBC arises from the improper selection of the SP2 calibration factor and mass loss occurring beyond the particle size detection range. The overestimation of eBC_AE_ can be attributed to several factors, including the effects of PM loading, multiple scattering caused by filters, and the default MAC provided by the manufacturer. Conversely, the overestimation of eBC pass-through (eBC_PASS_) is influenced by light-absorbing gases like NO_2_.

The complexity of the chemical composition in ambient samples, BB aerosols, etc., is more pronounced than in the aforementioned samples, leading to increased quantitative uncertainty in eBC measurements and a greater disparity between eBC and rBC. In a study by Raatikainen et al. [167] on ambient samples from the Finnish Arctic, comparisons were made between eBC_MAAP_ and rBC_SP2_. Surprisingly, the study revealed that eBC was five times higher than rBC, a significant difference that cannot be solely attributed to instrumental uncertainties. This phenomenon can be attributed to three main factors. Firstly, the b_abs_ values obtained by the optical instrument may encompass contributions from BrC or highly volatile light-absorbing carbon (LAC), which exhibits low light absorption at longer wavelengths and are considered non-refractory components. Consequently, these components cannot be detected with the SP2 instrument. Additionally, the AAE of BC internally mixed with non-absorbing material is 1.2 at wavelength of 440 nm, indicating a significant presence of coatings that do not absorb light on BC [212]. This suggests that the overestimation of eBC is also influenced by the contribution of non-absorbing coatings. Furthermore, although the Arctic generally exhibits good air quality with a scarcity of large-sized BC particles, the presence of BC particles smaller than 75 nm should not be overlooked. The particle size range detected with SP2 (calibration material is size-selected Aquadag^®^ particles) is concentrated in 75 to 655 nm, which leads to an underestimation of other-sized BC mass. Thirdly, the determination of default MAC values for optical instruments and the calibration materials used for SP2 is heavily dependent on the aerosol chemical composition, introducing significant uncertainty. Including non-refractory materials with BC, for example, can substantially increase the MAC of BC, resulting in an overestimation of eBC [213,214,215]. Additionally, using Fullerene Soot as a calibration material for SP2 produces results approximately 33% higher than those obtained with Aquadag [165,171,172], while this study employs Aquadag as the calibration material. These findings were also corroborated by a study conducted in the Canadian Arctic, where eBC_AE_/rBC_SP2_ remained constant at 2.7 throughout the observation period [192]. And the choice of b_abs_ correction scheme for filter-based optical instruments also contributes to the discrepancy between eBC and rBC measurements. For instance, Laing et al. [154] assessed BB aerosols and non-BB aerosols using AE and SP2, respectively, and discovered that eBC was 2.1 times higher than rBC due to the implementation of a smaller multiple scattering correction factor (1.57) in AE [131]. After correcting for this discrepancy, the appropriate correction factor was found to be 3.31. Similarly, Holder et al. [153] conducted measurements on BB aerosols in both field and laboratory settings, finding that eBC_AE_ values were 1.98 to 2.57 times higher than rBC_SP2_ values. Hence, it can be concluded that, in general, eBC exceeds rBC in terms of magnitude.

## 6. Conclusions and Prospects

**Figure 5:**
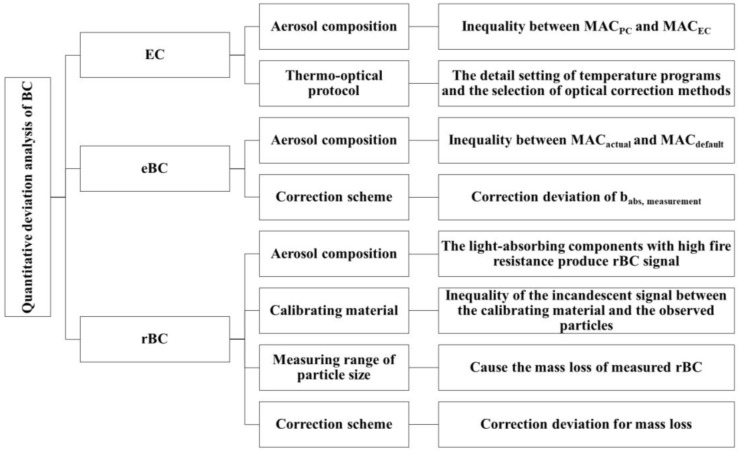
Quantitative deviation analysis of BC using different techniques.

**Figure 6:**
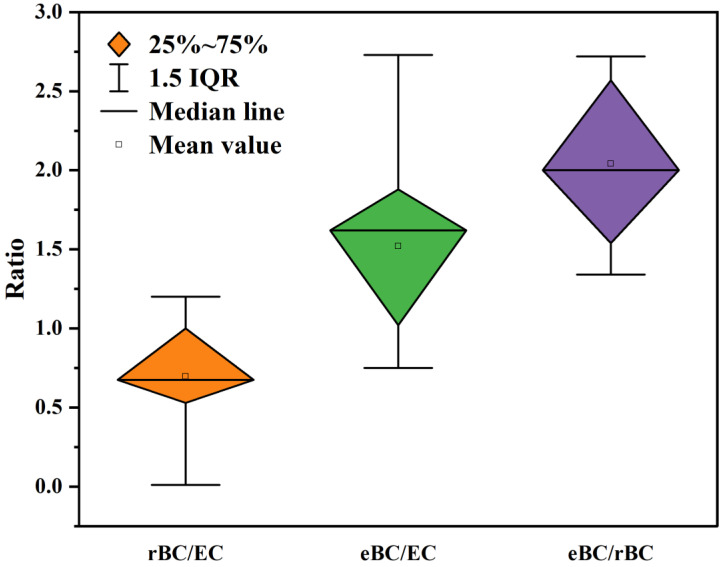
Comparison of BC quantitative results from the different techniques [28,31,90,152,153,154,157,165,173,192,193,194,199,202,204,206,208,210,211].

BC is a significant hazard to human health, the ecological environment, and climate change, garnering considerable attention in recent years. However, the mass concentration of BC obtained using different methods is very different, which not only interferes with the quantitative observation and comparison of BC, but also seriously hinders the accurate evaluation of the harmful effects of BC. Hence, this review systematically examines the detection principles, commercial instruments, bias factors, and data calibration schemes of three common BC quantification techniques: the TOA, optical, and LII techniques. Furthermore, it discusses the discrepancies in response among the three methods under varying atmospheric conditions. We have identified the factors that contribute to quantitative deviations in eBC, rBC, and EC (Figure 5). In essence, changes in MAC caused by BC aging are the most influential source of eBC bias, and we should not fully trust the reference MAC value provided by the manufacturer when using the light absorption measurement instruments. In addition, the light absorption measurement based on the filter method is easily affected by the multiple scattering effects of the filter and particles, which needs further correction. The pyrolysis of OC significantly contributes to the uncertainty of EC, while overestimation or underestimation of EC due to PC depends on the relative magnitude of MAC_PC_ and MAC_EC_. Therefore, if the OC component can be effectively removed before the use of TOA for quantification of EC, such as solvent extraction, this will improve the accuracy of EC quantification. LII technology is easily affected by the particle size detection range in the process of quantifying rBC, sometimes causing rBC underestimation. Therefore, when we use LII technology, we should choose appropriate calibration substances, precise built-in parameter settings, and reasonable data correction schemes. By summarizing the BC quantitative data mentioned in the literature, we have established a general rule that applies to most atmospheric conditions, i.e., eBC > EC > rBC (Figure 6). Future research endeavors in BC quantification ought to address three key aspects: 1. Investigating the influence mechanisms and contribution levels of non-BC components on MAC within different aerosol types. 2. Developing a sample pretreatment scheme that ensures zero loss of BC while effectively eliminating non-BC constituents before quantification. 3. Broadening the scope of BC observations under diverse atmospheric conditions by employing multiple co-located instruments, thereby enhancing the comparability of BC observation outcomes between regions.

## Figures and Tables

**Table 1:** Abbreviations, symbols, and units used in this paper.

Name	Abbreviation	Symbol	Unit
Absorption Ångström Exponent	AAE	-	
Aerosol Characterization Experiments	ACE	-	-
Aerosol Mass Spectrometer-Scanning Mobility Particle Sizer	AMS-SMPS	-	-
Aethalometer	AE	-	-
Babs produced by PM and filter	-	b_pf_	m^−1^
Biomass burning	BB	-	-
Black carbon	BC	-	-
Brown carbon	BrC	-	-
California Regional PM_10_/PM_2.5_ Air Quality Study	CRPAQS	-	-
Canadian National Air Pollution Surveillance	NAPS	-	-
Carbonate carbon	CC	-	-
Chemical Speciation Network	CSN	-	-
Collection area of the filter	-	A	m^2^
Collection efficiency	CE	-	%
Combustion Aerosol Standard	CAST	-	-
Continuous Soot Monitoring System	COSMOS	-	-
Correction factor for multiple scattering effects	-	C_ref_	-
Diameter	-	D	μm
EC defined by reflected light signal	EC_R_	-	μg C/cm^2^
EC defined by transmitted light signal	EC_T_	-	μg C/cm^2^
EC quantified by the protocol with an inert peak temperature is 870 °C	EC_870_	-	μg C/cm^2^
Elemental carbon	EC	-	μg C/cm^2^
Equivalent black carbon	eBC	-	μg/m^3^
European Supersites for Atmospheric Aerosol Research	EUSAAR	-	-
Extinction coefficient	-	b_ext_	Mm^−1^
Extinction minus scattering	EMS	-	-
High resolution-particle time-of-flight aerosol mass spectrometer	HR-ToF-AMS	-	-
Humic-like substances	HULIS	-	-
Inert peak temperature	T_peak_	-	°C
Inert peak temperature is 870 °C	T_peak_-870 °C/He-870	-	-
Insoluble organic carbon	ISOC	-	-
Integrating plate	IP	-	-
Integrating sandwich	IS	-	-
Interagency Monitoring of Protected Visual Environments	IMPROVE	-	-
Irradiance	-	I	MW/cm^2^
Laser-induced incandescence	LII	-	-
Light absorbing carbon	LAC	-	-
Light absorption coefficient	-	b_abs_	Mm^−1^
Light intensity	-	I	W/m^2^
Lower detection limit	LDL	-	nm
Mass	-	M	μg
Mass absorption cross-section	MAC	-	m^2^/g
Mixing state	-	D_p_/D_BC_	-
Multi-Angle Absorption Photometer	MAAP	-	-
National Institute for Occupational Safety and Health	NIOSH	-	-
Non-refractory particles	NR-PM	-	-
Organic aerosol	OA	-	-
Organic carbon	OC	-	μg C/cm^2^
Particle Soot Absorption Photometer	PSAP	-	-
Particulate matter	PM	-	μg/m^3^
Photo thermal interferometry	PTI	-	-
Photoacoustic Extinctiometer	PAX	-	-
Photoacoustic spectrometer	PAS	-	-
Pyrolytic carbon	PC	-	μg C/cm^2^
Reflected light	R	-	mv (millivolts)
Refractory black carbon	rBC	-	μg/m^3^
Refractory particles	R-PM	-	-
Relative humidity	-	RH	%
Sampling time	-	∆t	s
Scattering coefficient	-	b_sca_	Mm^−1^
Secondary organic aerosol	SOA	-	-
Single particle soot photometer	SP2	-	-
Single scattering albedo	SSA	ω	-
Soot particle–aerosol mass spectrometer	SP-AMS	-	-
Southeastern Aerosol Research and Characterization network	SEARCH	-	-
Speciation Trends Network	STN	-	-
Stage 4 of the inert mode	He4	-	-
Teflon-coated glass fiber	TFE	-	-
Temperature	-	T	°C
Thermal optical analysis	TOA	-	-
Thermal optical reflectance method	TOR	-	-
Thermal optical transmittance method	TOT	-	-
Thickness of the filter	-	X	m
Total Carbon	TC	-	μg C/cm^2^
Transmitted light	T	-	mv (millivolts)
Transmitted light attenuation	ATN	-	-
Tricolor Absorption Photometer	TAP	-	-
Upper detection limit	UDL	-	nm
Velocity of the gas passing through the filter	-	V	m^3^/s
Volatile organic compounds	VOCs	-	-
Water-soluble organic carbon	WSOC	-	-
Wavelength	-	λ	nm

**Table 2:** Standard commercial instruments for thermo-optical analysis (TOA).

Technical Classification	Instrument Model	Principle	Deviation Source	Reference
Offline	DRI thermal/optical reflectance carbon analyzer	OC and EC are separated sequentially under different temperatures and atmospheres, and optical correction is used to monitor the formation and evolution of PC in real time.	1. Selection of thermo-optical protocols; 2. Interference of non-EC chemical components in PM	[52]
	DRI Model 2001			[53]
	DRI Model 2015			[54]
	Sunset model 5 L analyzers			
Semi-continuous	Model RT-4			[55]

**Table 3:** Basic operation parameters of three common thermal–optical protocols.

		IMPROVE ^a^		IMPROVE_A ^a^		NIOSH5040 ^b^		NIOSH870 ^b^		EUSAAR_1 ^b^		EUSAAR_2 ^b^	
Step	Gas	Temperature (°C)	Time (s)	Temperature (°C)	Time (s)	Temperature (°C)	Time (s)	Temperature (°C)	Time (s)	Temperature (°C)	Time (s)	Temperature (°C)	Time (s)
OC1	Pure He	120	150–580	140	150–580	250	60	310	80	200	120	200	120
OC2	Pure He	250	150–580	280	150–580	500	60	475	80	300	150	300	150
OC3	Pure He	450	150–580	480	150–580	650	60	615	80	450	180	450	180
OC4	Pure He	550	150–580	580	150–580	850	90	870	110	650	180	650	180
EC1	2%O_2_ + 98%He	550	150–580	580	150–580	650	30	550	45	550	240	500	120
EC2	2%O_2_ + 98%He	700	150–580	740	150–580	750	30	625	45	850	150	550	120
EC3	2%O_2_ + 98%He	800	150–580	840	150–580	825	30	700	45	n/a	n/a	700	70
EC4	2%O_2_ + 98%He	n/a	n/a	n/a	n/a	920	>120	775	45	n/a	n/a	850	80
EC5	2%O_2_ + 98%He	n/a	n/a	n/a	n/a	n/a	n/a	850	45	n/a	n/a	n/a	n/a
EC6	2%O_2_ + 98%He	n/a	n/a	n/a	n/a	n/a	n/a	870	110	n/a	n/a	n/a	n/a

^a^ Residence time is flexible. ^b^ Advance from one temperature to the next one when a well-defined carbon peak has evolved.

**Table 4:** The effect mechanism of the chemical components in the PM on the quantification of EC.

Chemical Composition	Specific Classification	Influence Mechanism	Reference
Chemical composition	Calcium carbonate, natural calcite	They form an EC-like carbon signal.	[80]
Organic carbon	Brown carbon	1. It has strong light absorption at short wavelengths and interferes with the laser signal; 2. it forms PC.	[81]
	Humic-like substances	1. They have strong thermal stability and can be evolved in the oxidation stage; 2. they form PC.	[82]
Metal	Metallic oxides	The release of O_2_ in the inert mode causes EC and/or PC to early evolution.	[83]
	Metal salts	1. They reduce the oxidation temperature of EC; 2. they can increase the charring degree of OC.	[84]
Inorganic salt	NH_4_HSO_4_	It can change the charring degree of OC.	[58]
	K^+^, Na^+^	They can change the combustion temperature of carbonaceous components.	[85]
Refractory oxygen-containing surface groups	CO_1_^+^, CO_2_^+^	They can catalyze the early evolution of EC.	[86]

**Table 5:** Solvent categories, operation steps, sample types, and OC removal efficiency of solvent extraction method.

Solvent Categories	Operation Steps	Sample Types	OC Removal Efficiency	Reference
Ultra-pure water	Put the filter on the glass sand core (the area is 1.5 cm^2^), pour the 100 mL ultra-pure water into the glass sand core.	ambient sample	28~79%	[99]
Ultra-pure water	The liquid soaked in the filter was extracted from the 30 mL bottle with a 5 mL disposable syringe, filtered (0.45 μm PTFE Filter head), and transferred to another 30 mL bottle.	ambient sample	-	[108]
Ultra-pure water	The filter is placed on the glass sand core (the diameter is 37 mm), the ultra-pure water is introduced into the glass sand core, and the water consumption is dynamically set according to the TC loading.	ambient sample	-	[100]
Ultra-pure water	-	marine aerosol	50~56%	[101]
Ultra-pure water	Ultrasonic extraction of samples with ultra-pure water for 30 min	ambient sample	53.80%	[102]
Ultra-pure water	After cleaning the filter with ultra-pure water (100 mL), the liquid is gradually dripped into the filter sand with a pipette until thoroughly wet and filtered in a vacuum. To prevent the porous diaphragm from fouling, a protective filter is inserted between the sample and the diaphragm.	ambient sample	28~55%	[78]
Ultra-pure water	Same as [98].	ambient sample	-	[103]
Methanol	The filter was immersed in methanol followed by ultrasonic extraction for 1 h.	wood burning smoke	92~98%	[104]
Methanol	Same as [108].	ambient sample	85%	[109]
Ultra-pure water			40%	
Methanol	Immerse the filter sample in methanol for 1 h.	ambient sample	89%	[110]
Ultra-pure water	Same as [104].	ambient sample	42 ± 18%	[105]
Methanol			76 ± 29%	
Methanol	The sample is placed between the two blank filters and put together on the glass sand core of the vacuum filter. Methanol is added to the filter three times to ensure that the retention time of methanol at the sand core is more than 1 h.	biomass burning sample	93 ± 3.8%	[106]
		ambient sample	79.3 ± 10%	
A mixture of dichloromethane, acetone, and hexane	The filter was immersed in dichloromethane, acetone, and hexane (volume ratio 2:4:4) for 1 h. The solvent mixture was changed after 30 min and stirred gently regularly.	ambient sample	81%	[62]
A mixture of ultra-pure water, dichloromethane, and acetone	Non-BC material is removed by a two-step method, and the sample with a diameter of 47 mm is placed on the funnel sand core. First, the funnel is injected slowly with 50~200 mL distilled water, retained for 30 min, and then discharged. Then the 60 mL 1:1 mixture is injected into the funnel retained for 30 min and then released.	ambient sample	-	[98]
		diesel vehicle emission		
Dichloromethane	-	marine engine emission	-	[97]
Ultra-pure water	The filter was dried at 180 °C for 1 h, and then extracted via vacuum funnel extraction.	marine engine emission	-	[107]
Toluene				

**Table 6:** Summary of standard commercial instruments using optical methods.

Technology Classification	Instrument Names	Principle	Coverage Wavelength Range (nm)	Source of Deviation	Reference
In situ	PASS (Photoacoustic Soot Spectrometer)	Photoacoustic technology	405, 532, 781	BC aging	[113]
	PAX (Photoacoustic Extinctiometer)		405, 532, 870		[116]
	MSS (Micro Soot Sensor)		808		[114]
	PTI (Photo thermal interferometry)		450, 880		[126]
	TAP (Tricolor Absorption Photometer)		365, 467, 528, 652		[127]
	DPAS (Differential Photoacoustic Spectrometer)		473, 532, 671		[117]
Filter-based	MAAP (Multi-Angle Absorption Photometer)	Quantitative mass according to the attenuation of transmitted light passing through the filter.	637	PM morphology	[115]
	AE (Aethalometer)		370, 470, 520, 590, 660, 880, 950	Loading effect of PM, multiple scattering effects of filter, scattering effect of PM	[118]
	PSAP (Particle Soot Absorption Photometer)		467, 530, 660		[119]
	COSMOS (Continuous Soot Monitoring System)		565	Charring of low volatile organic compounds	[120]
	CLAP (Continuous Light Absorption Photometer)		467, 528, 652	BC aging	[121]

**Table 7:** Several common calibration schemes for filter-based methods.

Correction Scheme	Corrected Bias Effect	Instrument	Reference
Weingartner (2003)	filter loading effects, filter multiple scattering effects	AE30	[137]
Arnott (2005)	filter loading effects, filter multiple scattering effects, loaded aerosol scattering effects	AE31	[133]
Schmid (2006)	filter loading effects, filter multiple scattering effects, loaded aerosol scattering effects	AE30	[135]
Virkkula (2007)	filter loading effects	AE16, AE30	[136]
Coen (2010)	filter loading effects, filter multiple scattering effects, loaded aerosol scattering effects	AE10, AE16, AE31	[134]
Virkkula (2010)	filter loading effects, loaded aerosol scattering effects	TAP	[119]
Ogren (2010)	filter loading effects, loaded aerosol scattering effects	TAP	[138]
Drinovec (2015)	filter loading effects	AE33	[131]
Kim (2018)	filter multiple scattering effects, loaded aerosol scattering effects	AE31, CLAP	[130]

**Table 8:** Commonly used instruments for testing refractory black carbon (rBC).

Technology Classification	Instruments Names	Principle	Detection Ability	Source of Deviation	Reference
Laser induced incandescence	SP2 (Single Particle Soot Photometer)	Laser induced incandescence method	Range of particle size: 65~600 nm	1.PM particle size detection range; 2. calibration material	[30]
	SP2-XR (Single Particle Soot Photometer–Extended Range)		Range of particle size: 50~800 nm		[160]
	LII 300 (laser-Induced Incandescence Instrument System)		Range of laser flux: 0.6~3.2 mI/mm^2^	Particles of different sizes would reach different peak temperatures at different times, bringing uncertainty to the effective peak temperature.	[158]
Single-particle aerosol mass spectrum	SP-AMS (Soot Particle Aerosol Mass Spectrometer)	Laser induced incandescence, mass spectrometry	Range of particle size: PM_1.0_	Collection efficiency	[37]

## Data Availability

The data underlying this article are available in the corresponding references.
